# A Simple, Efficient, and Eco-Friendly Method for the Preparation of 3-Substituted-2,3-dihydroquinazolin-4(1*H*)-one Derivatives

**DOI:** 10.3390/molecules24224052

**Published:** 2019-11-09

**Authors:** Zainab Almarhoon, Kholood A. Dahlous, Rakia Abd Alhameed, Hazem A. Ghabbour, Ayman El-Faham

**Affiliations:** 1 Department of Chemistry, College of Science, King Saud University, P.O. Box 2455, Riyadh 11451, Saudi Arabia; zalmarhoon@ksu.edu.sa (Z.A.); kdahloos@ksu.edu.sa (K.A.D.); Roki.ahmed@yahoo.com (R.A.A.); 2 Department of Medicinal Chemistry, Faculty of Pharmacy, University of Mansoura, Mansoura 35516, Egypt; ghabbourh@yahoo.com; 3 Chemistry Department, Faculty of Science, Alexandria University, P.O. Box 426, Ibrahimia, Alexandria 12321, Egypt

**Keywords:** 2-MeTHF, isatoic anhydride, X-ray single crystals, 2,3-dihydroquinazolin-4(1*H*)-one, microwave irradiation

## Abstract

A simple, cost-effective method under environmentally benign conditions is a very important concept for the preparation of 2,3-dihydroquinazolin-4(1*H*)-one derivatives. The present work describes an efficient and eco-friendly protocol for the synthesis of 2-amino-*N*-(2-substituted-ethyl)benzamide and 3-substituted-2,3-dihydroquinazolin-4(1*H*)-one derivatives. The novel feature of this protocol is the use of 2-methyl tetrahydrofuran (2-MeTHF) as an eco-friendly alternative solvent to tetrahydrofuran (THF) in the first step. In the second step, methanol in the presence of potassium carbonate as a catalyst was used under conventional heating or microwave irradiation, which provided an eco-friendly method to afford the target products in excellent yields and purities. NMR (^1^H and ^13^C), elemental analysis, and LC-MS confirmed the structures of all compounds. X-ray crystallography further confirmed the structure of the intermediate 2-amino-*N*-(2-substituted-ethyl)benzamide **3a**. The molecular structure of **3a** was monoclinic crystal, with *P*21/*c*, *a* = 13.6879 (11) Å, *b =* 10.2118 (9) Å, *c* = 9.7884 (9) Å, *β* = 105.068 (7)°, *V* = 1321.2 (2) Å3, and *Z* = 4.

## 1. Introduction

The dihydroquinazolinone moiety is present in numerous biologically active molecules with various potential therapeutic effects [1,2,3,4,5,6,7,8,9]. In view of the increasingly widespread applications of dihydroquinazolinone derivatives, many researchers have made great effort to develop these types of compounds using different synthetic methodologies [8,10,11,12,13,14,15,16,17,18,19,20,21,22,23,24], such as by using TiO_2_ nanoparticles (TiO_2_ NPs) as a catalyst for the synthesis of 2,3-disubstituted dihydroquinazolin-4(1*H*)-one derivatives [25]; a metal reduction–condensative cyclization strategy [26]; a three-component reaction of isatoic anhydride with amine and 2-formyl benzoic acid in the presence of montmorillonite K10 as a catalyst [27]; ultrasound irradiation catalyzed by dodecylbenzenesulfonic acid [28]; microwave irradiation in the presence of Amberlyst-15 [29] or Cu-CNTs [30]; and condensation of isatoic anhydride, amine, or ammonium salts with aldehydes or ketones in the presence of *p*-toluenesulfonic acid [31], citric acid [32], or silica bonded with different acids [33,34,35]. Moreover, the development of a simple, efficient, easy method under environmentally benign conditions for the synthesis of 2,3-dihydroquinazolin-4(1*H*)-one derivatives is necessary.

Herein, we report an efficient and eco-friendly protocol for the synthesis of 2-amino-*N*-(2-substituted-ethyl)benzamide from isatoic anhydride using 2-methyltetrahydrofuran (2-MeTHF), which has previously been promoted as an eco-friendly alternative solvent to tetrahydrofuran (THF) [36,37,38]. In the second step, 2-aminobenzamide intermediate was reacted with different aldehydes under conventional heating or microwave irradiation (MWI) in the presence of methanol as a solvent and K_2_CO_3_ as a catalyst to afford the new 2,3-dihydroquinazolin-4(1*H*)-one derivatives.

## 2. Results and Discussion

**Scheme 1:**
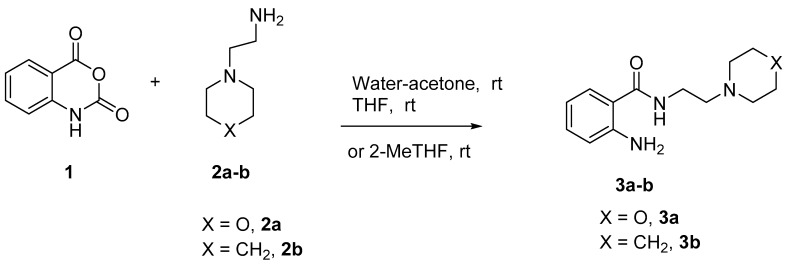
Synthesis of 2-amino-*N*-(2-substituted-ethyl)benzamide.

The target products were obtained via two simple and eco-friendly protocols. First, to select the best solvent and conditions for the reaction of isatoic anhydride **1** with two biologically active amine moieties **2a** and **2b** [39,40,41], 2-morpholinoethan-1-amine **2a** was reacted with **1** in different solvents water–acetone [42], THF, and 2-MeTHF [36,37,38] in order to compare the yield, purity, and time required to afford the final product **3a** (Scheme 1). The three methods worked satisfactorily, but using the 2-MeTHF gave higher yield and purity in somewhat less reaction time, as summarized in Table 1. 

In 1951, Bost et al. [43] reported the synthesis of **3a** via the reaction of ethyl-2-aminobenzoate with **2a**. Later, in 1970, Bonola et al [44] reported the synthesis of **3b** as a key intermediate for the synthesis of 2,3-dihydro-4(1*H*)-quinazolinone derivatives. Recently, Li et al. [41] reported the synthesis of **3a** and **3b** without isolation of the product, and they used an in situ reaction for the preparation of thioxodihydroquinazolinone derivatives. Herein we report the synthesis of **3a** and **3b** under eco-friendly conditions at room temperature to give the products in high yields and purities. 

**Figure 1:**
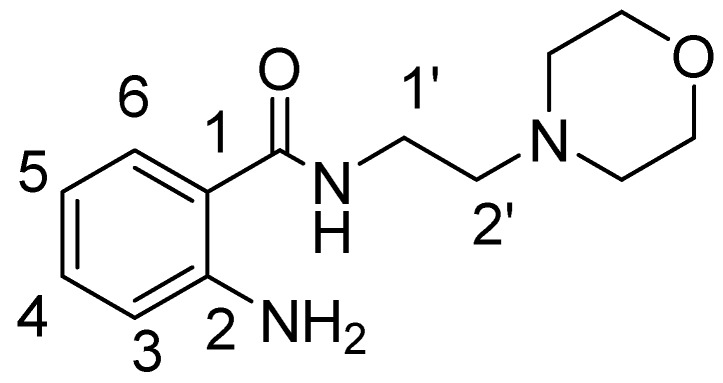
Structure of **3a**.

The structure of **3a** (Figure 1) was confirmed by the ^1^H-NMR spectrum (Figure S1, Supporting information) which showed a broad singlet peak at δ 2.55 related to four protons (CH_2_NCH_2_, morpholine), a triplet at δ 2.63 (CH_2_N, *H_2′_*), a triplet at δ 3.53 (CH_2_N, *H_1′_*), a broad singlet peak at δ 3.74 related to four protons (CH_2_OCH_2,_ morpholine), a singlet at δ 5.50 related to NH, a multiplet at δ 6.62–6.66 for the two aromatic protons (*H_3_*, *H_5_*), a broad singlet at δ 6.82 for the NH, a triplet at δ 7.17(*H_4_*), and a doublet at δ 7.34(*H_6_*). The ^13^C-NMR spectrum for **3a** showed peaks at δ 35.5(*C_2′_*), 53.3(C–N–C, morpholine), 57.1(*C_1′_*), 66.6 (C–O–C, morpholine), 116.0(*C_3_*), 116.6(*C_1_*), 117.3(*C_5_*), 127.3(*C_6_*), 132.3(*C_4_*), 148.7(*C_2_*), and 169.3(CON) ppm.

**Figure 2:**
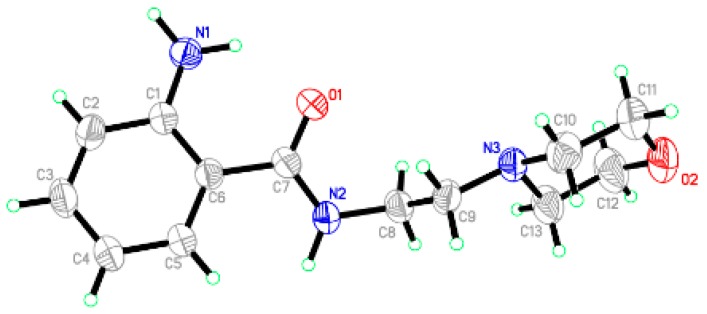
Oak Ridge Thermal Ellipsoid Plot (ORTEP)diagram of the titled compound; displacement ellipsoids are plotted at the 40% probability level for non-H atoms.

**Figure 3:**
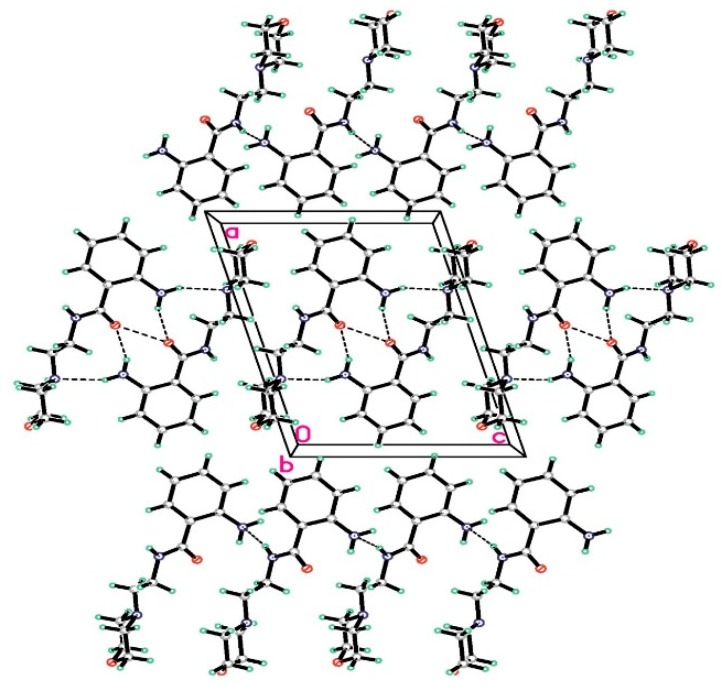
Molecular packing of the titled compound displaying hydrogen bonds, which are drawn as dashed lines along the *c*-axis.

The structure of **3a** was further confirmed by the X-ray single crystal differaction technique (Figure 2). The crystallographic data and refinement information for **3a** are summarized in Table 2. The selected bond lengths and bond angles are listed in Table S1 (Supporting information). The asymmetric unit contains one independent molecule, as shown in Figure 2. All the bond lengths and angles are in the normal ranges [45]. In the crystal packing (Figure 3), molecules are linked via three classical and one non-classical intermolecular hydrogen bonds (Table S2, supporting information).

Demonstrating the applicability of the method used, the **3b** was obtained in high yield and purity as observed from its spectral data (Figure S2, Supporting information) using the same previous conditions. 

Compounds **3a** and **3b** were reacted with different aldehydes using methanol in the presence of K_2_CO_3_ as a catalyst under conventional heating and microwave irradiation (Scheme 2, Table 3). Microwave irradiation (MWI) afforded the products in less reaction time with higher yields and purities without further purification, as indicated from their spectral data (Figures S3–S13, Supporting information).

**Figure 4:**
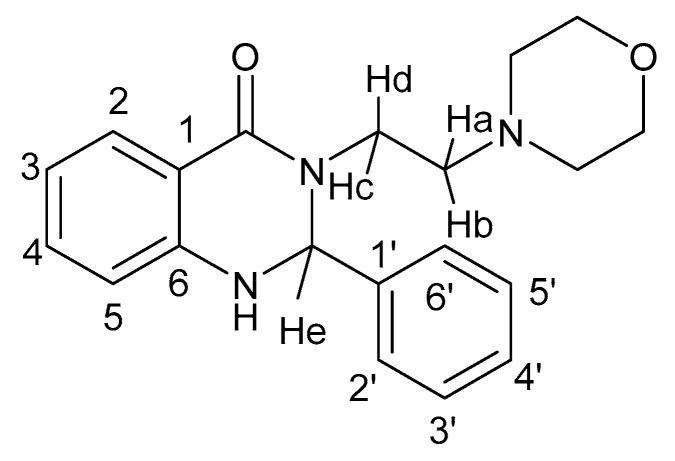
Structure of **4a**.

^1^H-NMR of **4a** in CDCl_3_ as a prototype showed a mutiplet peak at δ 2.40–2.46 related to the four protons of the morpholine residue (CH_2_NCH_2_) and the diastereotopic proton *Ha,* where it has two couplings with *Hb* and the other two protons *Hc* and *Hd* (Figure 4). The multiplet peak observed at δ 2.60 corresponds to the second diastereotopic proton *Hb* due to the coupling with *Ha*, *Hc* and *Hd*. The same observation was noticed for the other two diastereotopic protons *Hc* and *Hd*, where two multiplet peaks were observed at δ 3.04 and 3.93, respectively. The other protons for the morpholine ring showed a triplet peak at δ 3.65 (CH_2_OCH_2_). The two singlet peaks at δ 4.50 and 5.90 correspond to *He* and NH, respectively. The doublet peak at δ 6.52 is related to *H5*, the triplet at δ 6.85 corresponds to *H_3_*, the triplet at δ 7.26 corresponds to *H_4_*, the multiplet at δ 7.35–7.38 corresponds to the five protons of the phenyl group, and the doublet peak at δ 7.94 is related to *H_2_*. The ^13^C-NMR spectrum of **4a** in CDCl_3_ showed peaks at δ 41.1 corresponding to –NCH_2_*C*H_2_N–, δ 53.2 related to *C*H_2_N*C*H_2_ (morpholine ring), δ 55.7 related to the carbon of the ethylene moiety (OC–N*C*H_2_CH_2_N–), δ 65.6 corresponding to *C*H_2_O*C*H_2_ (morpholine), and δ 57.0 corresponding to N*C*HN (quinazolinone ring). The aromatic carbon showed peaks at δ 114.7 (*C_5_*), 115.7 (*C_1_*), 119.0 (*C_3_*), 126.6 (*C_2′_*_,*4′*,*6′*_), 128.24 (*C_2_*), 128.9, 129.2 (*C_3′_*_,*5′*_), 133.7 (*C_4_*), 140.0 (*C_1′_*), and 148.4 (*C_6_*), while the peak at δ 163.6 corresponds to the carbonyl group (C=O).

Since the formation of the cyclic compounds, 3-substituted-2,3-dihydroquinazolin-4(1*H*)-one derivatives **4**–**6,** create a new chiral center on the final product, it is expected that this will lead to the formation of two enantiomers *R* and *S* as a racemic mixture. 

**Scheme 2:**
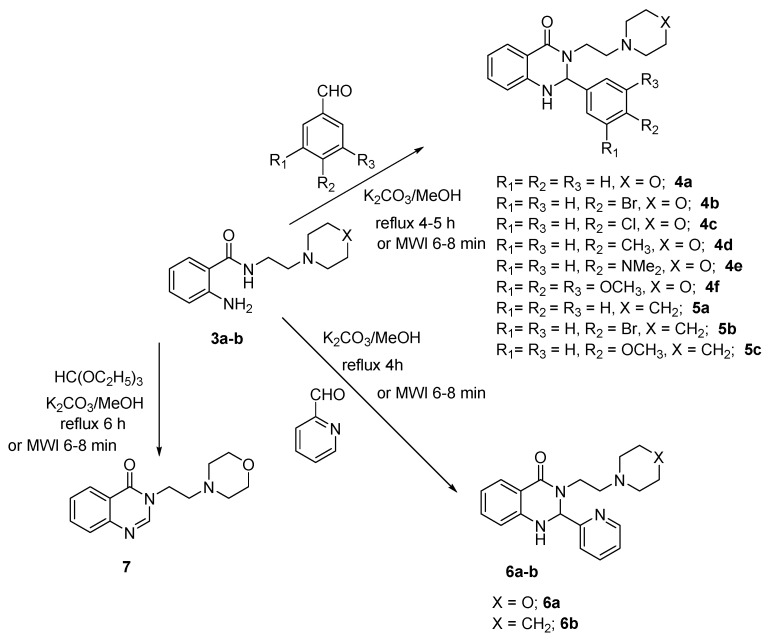
Synthesis of 3-substituted-2,3-dihydroquinazolin-4(1*H*)-one derivatives.

Compound **7** (Scheme 2) has been reported previously by Xu et al. [46] who, using CuI/4-hydroxy-L-proline, catalyzed the reaction of *N*-substituted o-bromobenzamides with formamide at 80 °C in DMF for 24 h. 

Herein, 3-(2-morpholinoethyl)quinazolin-4(3*H*)-one **7** was obtained in higher yield and purity, as observed from its spectral data (Figure S14, Supporting information) under the same conditions described above.

## 3. Materials and Methods 

All reagents and solvents were purchased from commercial suppliers and were used without further purification. ^1^H-NMR and ^13^C-NMR spectra were recorded on a JEOL 400 MHz spectrometer (JEOL, Ltd, Tokyo, Japan), and chemical shift (*δ*) values are expressed in ppm. Mass spectra were recorded on a JEOL JMS-600 H (JEOL, Ltd, Tokyo, Japan). Elemental analyses were carried out on an Elmer 2400 CHNS Elemental Analyzer (PerkinElmer, Inc.940 Winter Street, Waltham, MA, USA). Melting points were measured on a Gallenkamp melting point apparatus (Sigma-Aldrich Chemie GmbH, 82024 Taufkirchen, Germany) in open glass capillaries and are uncorrected. Experiments were performed in a multimode reactor (Synthos 3000, Aton Paar GmbH, 1400 W maximum magnetron, Germany). The vessel was purged with nitrogen gas for 1 min and then placed in the corresponding rotor fixed with a screw; the rotor was then closed with a protective hood. After heating for 6–8 min (600 W at 60 °C), cooling was accomplished by a fan for 5 min.

### 3.1. General Method for the Synthesis of 2-amino-N-(2-substituted-ethyl)benzamide

To a solution of isatoic anhydride (1.63 gm, 10 mmol) in 2Me-THF (50 mL) was added 2-(piperidin-1-yl)ethan-1-amine or 2-morpholinoethan-1-amine (11 mmol). The reaction mixture was stirred at room temperature for 4–5 h (TLC showed complete reaction after 4 h). 2Me-THF was evaporated under reduced pressure to afford the target products in good yields and purities as observed form their spectral data. Compound **3a** was obtained as single crystals by slow evaporation at room temperature from dichloromethane–hexane (4:6).

*2-Amino-N-(2-morpholinoethyl)benzamide* (***3a****).* Light brown crystals in 95% yield; mp 126–128 °C (lit [43] mp 126 °C); ^1^H-NMR (CDCl_3_): δ 2.55 (4H, br.s, CH_2_NCH_2_), 2.63 (2H, t, *J* = 6 Hz, CH_2_CH_2_N), 3.53 (2H, t, *J* = 2.8, 2 Hz, NHCH_2_CH_2_N ), 3.74 (4H, br.s, CH_2_OCH_2_), 5.50 (2H, br.s, NH_2_), 6.62–6.66 (2H, m, Ar), 6.82 (1H, br.s, NH), 7.17 (1H, t, *J* = 7.2 Hz, Ar), 7.34 (1H, d, *J* = 8 Hz, Ar) ppm; ^13^C-NMR (CDCl_3_): δ 35.5, 53.3, 57.1 ,66.6, 116.0, 116.6, 117.3, 127.3, 132.3, 148.8, 169.3 ppm. LC/MS (ESI): 250.12 [M + H]^+^; Anal. for C_13_H_19_N_3_O_2_; Calcd: C, 62.63; H, 7.68; N, 16.85; Found: C, 62.88; H, 7.87; N, 17.01.

*2-Amino-N-(2-(piperidin-1-yl)ethyl)benzamide **3b***. Light brown crystals in 96% yield; mp 113–114 °C; (lit [44] yield 81%, mp 130–132 °C from benzene); ^1^H-NMR (CDCl_3_): δ 1.45 (2H, m, CH_2_), 1.61 (4H, td, *J* = 4.8, 6, 5.2, 6 Hz, 2CH_2_), 2.46 (4H, br.s, CH_2_NCH_2_), 2.57 (2H, t, *J* = 5.6, 6 Hz, CH_2_N), 3.49 (2H, q, *J* = 6, 5.2, 4.8 Hz, CH_2_N), 5.53 (2H, br.s, NH_2_), 6.63–6.66 (2H, m, Ar), 6.95 (1H, br.s, NH), 7.19 (1H, t, *J* = 8, 6.4 , 1.6 Hz, Ar), 7.35 (1H, dd, *J* = 8, 6.4, 1.6 Hz, Ar) ppm; ^13^C-NMR (CDCl_3_): δ 24.2, 25.8, 36.0, 54.2, 57.0, 116.3, 116.5, 117.1, 127.3, 132.0, 148.6, 169.2 ppm. LC/MS (ESI): 248.12 [M + H]^+^; Anal. for C_14_H_21_N_3_O; Calcd: C, 67.98; H, 8.56; N, 16.99; Found: C, 68.08; H, 8.69; N, 17.03.

### 3.2. General Method for the Synthesis of 3-substituted-2,3-dihydroquinazolin-4(1H)-one Derivatives 

Method A—Conventional heating: A mixture of 2-amino-*N*-(2-substituted-ethyl)benzamide **3a**/**b** (1 mmol), benzaldehyde derivatives or 2-pyridine carboxaldehyde (1 mmol), and potassium carbonate (1.3 mmol) in MeOH (5 mL) was refluxed for 4–6 h. After completion of the reaction, potassium carbonate was filtered off from the hot solution and washed with hot methanol (10 mL). Pure product was obtained on the cooling and evaporation of methanol at room temperature.

Method B—Microwave irradiation**:** A mixture of 2-amino-*N*-(2-substituted-ethyl)benzamide **3a**/**b** (1 mmol), benzaldehyde derivatives or 2-pyridine carboxaldehyde (1 mmol), and potassium carbonate (1.3 mmol) in MeOH (5 mL) was mixed at RT and then microwave irradiated (600 W and 60 °C) using a Galanz microwave oven (Guangdong Galanz Enterprise Co, ltd. China) or a multimode reactor for 6–8 min. After cooling, hot methanol was added, and potassium carbonate was filtered off from the solution and washed with hot methanol (10 mL). Pure product was obtained in excellent yield and purity after the evaporation of methanol at room temperature.

*3-(2-Morpholinoethyl)-2-phenyl-2,3-dihydroquinazolin-4(1H)-one* (**4a**). Light brown solid in 86% (Method A) or 92% (Method B) yield; mp 142–144 °C (lit [44] yield 30%, mp 144–147 °C); ^1^H-NMR (CDCl_3_): δ 2.40–2.46 (5H, m), 2.60 (1H, m), 3.04 (1H, m), 3.65 (4H, t, *J* = 4.4 Hz, CH_2_OCH_2_), 3.93 (1H, m), 4.50 (1H, s, HNCHN), 5.90 (1H, s, NH), 6.52 (1H, d, *J* = 8 .0 Hz, Ar), 6.85 (1H, d, *J* = 7.6 Hz, Ar), 7.26 (1H, d, *J* = 7.6 Hz, Ar), 7.35–7.38 (5H, m, phenyl), 7.94 (1H, dd, *J* = 8.0, 1.2 Hz, Ar) ppm; ^13^C-NMR (CDCl_3_): δ 41.1, 53.2, 55.7, 65.6, 57.0, 114.7, 115.7, 119.0, 126.6, 128.2, 128.9, 129.1, 133.7, 140.0, 148.4, 163.6 ppm. LC/MS (ESI): 338.12 [M + H]^+^; Anal. for C_20_H_23_N_3_O_2_; Calcd: C, 71.19; H, 6.87; N, 12.45; Found: C, 71.33; H, 6.69; N, 12.23.

*2-(4-Bromophenyl)-3-(2-morpholinoethyl)-2,3-dihydroquinazolin-4(1H)-one* (**4b**). Light brown solid in 87% (Method A) or 96% (Method B) yield; mp 165–166 °C; ^1^H-NMR (CDCl_3_): δ 2.43 (4H, br.s, CH_2_NCH_2_), 2.49 (1H, m), 2.62 (1H, m), 3.01 (1H, m), 3.66 (4H, br.s, CH_2_OCH_2_), 3.99 (1H, m), 4.52 (1H, s, CH), 5.88 (1H, s, NH), 6.52 (1H, d, *J* = 8 Hz, Ar), 6.84 (1H, d, *J* = 7.6 Hz, Ar), 7.24 (3H, m, Ar), 7.45 (2H, d, *J* = 6.4 Hz, Ar_`_), 7.92 (1H, dd, *J* = 8, 1.6 Hz, Ar) ppm; ^13^C-NMR (CDCl_3_): δ 40.4, 52.6, 55.1, 64.3, 71.8, 115.1, 119.5, 123.6, 127.7, 127.9, 132.1, 132.4, 134.2, 138.5, 144.9, 163.6 ppm. LC/MS (ESI): 416.12 [M + H]^+^; Anal. for C_20_H_22_BrN_3_O_2_; Calcd: C, 57.70; H, 5.33; N, 10.09; Found: C, 57.89; H, 5.47; N, 10.26.

*2-(4-Chlorophenyl)-3-(2-morpholinoethyl)-2,3-dihydroquinazolin-4(1H)-one* (**4c**). Light brown solid in 84% (Method A) or 96% (Method B) yield; mp 133–135 °C; ^1^H-NMR (CDCl_3_): δ 2.43 (4H, br.s, CH_2_NCH_2_), 2.49 (1H,m), 2.62 (1H, m), 3.00 (1H, m), 3.66 (4H, br.s, CH_2_OCH_2_), 3.99 (1H, m), 4.52 (1H, s, CH), 5.88 (1H, s, NH), 6.52 (1H, d, *J* = 8 Hz, Ar), 6.84 (1H, t, *J* = 7.6 Hz, Ar), 7.24 (3H, m, Ar), 7.45 (2H, d, *J* = 6.8 Hz, Ar), 7.92 (1H, dd, *J* = 8.0, 1.6 Hz, Ar) ppm. ^13^C-NMR (CDCl_3_): δ 40.6, 52.6, 54.7, 64.0, 70.5, 115.4, 118.9, 127.78, 128.1, 128.9, 134.1, 134.5, 138.9, 145.5, 164.0 ppm. LC/MS (ESI): 372.12 [M + H]^+^; Anal. for C_20_H_22_ClN_3_O_2_; Calcd: C, 64.60; H, 5.96; N, 11.30; Found: C, 64.78; H, 6.12; N, 11.54.

*3-(2-Morpholinoethyl)-2-(p-tolyl)-2,3-dihydroquinazolin-4(1H)-one* (**4d**). Off-white solid in 83% (Method A) or 96% (Method B) yield; mp 132–134 °C; ^1^H-NMR (CDCl_3_): δ 2.33 (3H, s, CH_3_), 2.52 (5H, br.s, CH_2_NCH_2_ and H ethylene), 2.70 (1H, m), 3.13 (1H, m), 3.71 (4H, t, *J* = 4.4 Hz, CH_2_OCH_2_), 3.94 (1H, m), 4.51 (1H, s, CH), 5.90 (1H, s, NH), 6.52 (1H, d, *J* = 8 Hz, Ar), 6.82 (1H, t, *J* = 7.2 Hz, Ar), 7.16 (2H, d, *J* = 6.8 Hz, Ar), 7.26–7.29 (3H, m, Ar), 7.92 (1H, dd, *J* = 7.6 Hz, Ar) ppm; ^13^C-NMR (CDCl_3_): δ 21.1, 41.4, 53.6, 56.3, 66.5, 72.7, 114.3, 116.0, 119.3, 126.7, 128.4, 129.6, 133.6, 136.7, 139.4, 145.2, 163.4 ppm. LC/MS (ESI): 352.33 [M + H]^+^; Anal. for C_21_H_25_N_3_O_2_; C, 71.77; H, 7.17; N, 11.96; Found: C, 71.92; H, 7.32; N, 11.77.

*2-(4-(Dimethylamino)phenyl)-3-(2-morpholinoethyl)-2,3-dihydroquinazolin-4(1H)-one* (**4e**). Yellow solid in 87% (Method A) or 96% (Method B) yield; mp 183–184 ^°^C; ^1^H-NMR (DMSO-d_6_): δ 2.31 (4H, br.s, CH_2_NCH_2_), 2.49 (2H, s, NCH_2_CH_2_N), 2.86 (6H, s, CH_3_NCH_3_), 3.50 (5H, t, *J* = 4.4 Hz, CH_2_OCH_2_ and H ethylene), 3.83 (1H, m), 4.51 (1H, s, CH), 5.76 (1H, s, NH), 6.59–6.66 (4H, m, Ar), 7.10–7.16 (3H, m, Ar), 7.61 (1H, dd, *J* = 7.6 Hz, Ar) ppm; ^13^C-NMR (DMSO-d_6_): δ 40.3, 41.0, 53.3, 55.9, 66.2, 70.7, 112.0, 114.1, 114.8, 116.8, 127.3, 127.4, 128.1, 133.1, 146.6, 150.6, 162.4 ppm. LC/MS (ESI): 381.39 [M + H]^+^; Anal. for C_22_H_28_N_4_O_2_; Calcd: C, 69.45; H, 7.42; N, 14.73; Found: C, 69.63; H, 7.55; N, 14.89.

*3-(2-Morpholinoethyl)-2-(3,4,5-trimethoxyphenyl)-2,3-dihydroquinazolin-4(1H)-one* (**4f**). Off-white solid in 86% (Method A) or 97% (Method B) yield; mp 148–150 °C; ^1^H-NMR (400 MHz, CDCl_3_): δ 2.40 (4H, t, *J* = 4.4, 5.2 Hz, CH_2_NCH_2_), 2.58 (1H, m), 3.13 (1H, m), 3.63 (6H, two s, 2OCH_3_), 3.77 (3H, s, OCH_3_), 3.80 (4H, br.s, CH_2_OCH_2_), 3.88–3.91 (2H, m, CONCH_2_CH_2_N), 4.46 (1H, s, CH), 5.80 (1H, s, NH), 6.53 (1H, d, *J* = 8 Hz, Ar), 6.59 (2H, s, Ar), 6.83 (1H, t, *J* = 7.4Hz, Ar), 7.23 (1H, t, *J* = 8 Hz, Ar), 7.92 (1H, dd, *J* = 8, 1.6 Hz, Ar) ppm; ^13^C-NMR (CDCl_3_): δ 41.5, 53.8,56.1, 56.5, 60.8, 66.9, 73.2, 103.9, 114.2, 115.9, 119.3, 128.3, 133.5, 135.1, 138.8, 145.3, 153.5, 163.3 (CON) ppm. LC/MS (ESI): 428.45 [M + H]^+^; Anal. for C_23_H_29_N_3_O_5_; Calcd: C, 64.62; H, 6.84; N, 9.83; Found: C, 64.87; H, 6.95; N, 10.02.

*2-Phenyl-3-(2-(piperidin-1-yl)ethyl)-2,3-dihydroquinazolin-4(1H)-one* (**5a**). Off-white solid in 80% (Method A) or 93% (Method B) yield; mp 132–134 °C (lit [44] yield 67% mp 139–143 °C); ^1^H-NMR (CDCl_3_): δ 1.43 (2H, br.s, CH_2_), 1.61 (4H, br.s, 2CH_2_), 2.50 (5H, br.s, 2CH_2_ and CH), 2.75 (1H, m, CH), 3.12 (1H, dd, *J* = 5.2 Hz, CH ), 4.02 (1H, m, CH), 4.58 (1H, s, CH), 5.96 (1H, s, NH), 6.51 (1H, d, *J* = 8 Hz, H_8_), 6.82 (1H, t, *J* = 7.2 Hz, H_6_), 7.24 (1H, t, *J* = 8.0 Hz, H_7_), 7.32–7.36 (5H, m, phenyl), 7.91 (1H, dd, *J* = 6.8 Hz, Ar) ppm; ^13^C-NMR (CDCl_3_): δ 23.8, 25.2, 54.5, 41.7, 56.4, 72.7, 114.3, 116.0, 119.2, 126.6, 128.4, 128.9, 129.3, 133.5, 139.9, 145.1, 163.2 (CON) ppm. LC/MS (ESI): 336.23 [M + H]^+^; Anal. for C_21_H_25_N_3_O; Calcd: C, 75.19; H, 7.51; N, 12.53; Found: C, 75.33; H, 7.68; N, 12.87.

*2-(4-Bromophenyl)-3-(2-(piperidin-1-yl)ethyl)-2,3-dihydroquinazolin-4(1H)-one* (**5b**). Off-white solid in 82% (Method A) or 97% (Method B) yield; mp 196–198 °C; ^1^H-NMR (CDCl_3_): δ 1.43 (2H, dd, *J* = 5.2, 5.6 Hz, CH_2_CH_2_CH_2_), 1.59 (4H, dd, *J* = 4.8, 5.2 Hz CH_2_CH_2_CH_2_), 2.46 (4H, br.s, CH_2_NCH_2_), 2.52–2.71 (2H, m, CH_2_), 3.01 (1H, m, CH), 4.05 (2H, m, CH), 4.75 (1H, s, CH), 5.94 (1H, s, NH), 6.53 (1H, d, *J* = 8 Hz, Ar ), 6.83 (1H, t, *J* = 8 Hz, Ar), 7.23 (2H, d, *J* = 8.8 Hz, Ar), 7.24 (1H, t, *J* = 8 Hz, Ar), 7.43 (2H, d, *J* = 8.8 Hz, Ar), 7.90 (1H, dd, *J* = 8, 1.8 Hz, Ar) ppm; ^13^C-NMR (CDCl_3_): δ 23.7, 25.2, 54.6, 41.8, 56.4, 71.8, 114.7,116.1, 119.5, 127.9, 128.4, 129.1, 133.7, 135.1, 138.6, 144.8, 163.1 (CON) ppm. LC/MS (ESI): 415.40 [M + H]^+^; Anal. for C_21_H_24_BrN_3_O; Calcd: C, 60.87; H, 5.84; N, 10.14; Found: C, 60.99; H, 5.93; N, 10.33.

*2-(4-Methoxyphenyl)-3-(2-(piperidin-1-yl)ethyl)-2,3-dihydroquinazolin-4(1H)-one* (**5c**). Off-white solid in 81% (Method A) or 94% (Method B) yield; mp 146–147 °C; ^1^H-NMR (CDCl_3_): δ 1.40 (2H, d, *J* = 5.6 Hz, CH_2_CH_2_CH_2_), 1.56 (4H, dd, *J* = 5.2, 5.6 Hz, CH_2_CH_2_CH_2_), 2.45 (4H, br. s, CH_2_NCH_2_), 2.48–2.65 (2H, m, CH_2_), 3.10 (1H, m, CH), 3.75 (3H, s, OCH_3_), 3.94 (1H, m, CH), 4.53 (1H, s, CH), 5.88 (1H, s, NH), 6.50 (1H, d, *J* = 8 Hz, Ar), 6.80 (1H, t, *J* = 8 Hz, Ar), 6.81 (2H, d, *J* = 8.8 Hz, Ar), 7.20 (1H, t, *J* = 7.6 Hz, Ar), 7.29 (2H, d, *J* = 8.8 Hz, Ar), 7.89 (1H, d, *J* = 7.6 Hz, Ar) ppm; ^13^C-NMR (CDCl_3_): δ 23.9, 25.5, 54.5, 41.5, 56.4, 55.3, 72.4, 114.2,114.3, 115.93, 119.1, 128.0, 128.3, 131.9, 133.4, 145.3, 160.3, 163.25 (CON) ppm. LC/MS (ESI): 366.53 [M + H]^+^; Anal. for C_22_H_27_N_3_O_2_; Calcd: C, 72.30; H, 7.45; N, 11.50; Found: C, 72.56; H, 7.66; N, 11.74.

*3-(2-Morpholinoethyl)-2-(pyridin-2-yl)-2,3-dihydroquinazolin-4(1H)-one* (**6a**). Off-white solid in 83% (Method A) or 97% (Method B) yield; mp 154–155 °C; ^1^H-NMR (CDCl_3_): δ 2.48 (4H, br.s, CH_2_NCH_2_), 2.63 (2H, m, CH_2_), 3.15 (1H, m, CH), 3.64 (4H, td, *J* = 5.2, 4.4 Hz, CH_2_OCH_2_), 4.23 (1H, m, CH ), 5.20 (1H, s, CH), 5.83 (1H, s, NH), 6.51 (1H, d, *J* = 8 Hz, Ar), 6.77 (1H, t, *J* = 7.2 Hz, Ar), 7.17–7.24 (3H, m, Ar), 7.58 (1H, d, *J* = Hz, Ar), 7.86 (1H, dd, *J* = 7.2 , 1.6 Hz, Ar), 8.55 (1H, dd, *J* = 4.4 Hz, Ar) ppm; ^13^C-NMR (CDCl_3_): δ 42.7, 53.7, 56.7, 66.9, 72.6, 114.8, 116.3, 119.3, 120.1, 123.3, 128.3, 133.5, 136.9, 145.2, 149.8, 159.2, 163.08 (CON) ppm. LC/MS (ESI): 340.16 [M + H]^+^; Anal. for C_19_H_22_N_4_O_2_; Calcd: C, 67.44; H, 6.55; N, 16.56; Found: C, 67.66; H, 6.68; N, 16.79.

*3-(2-(Piperidin-1-yl)ethyl)-2-(pyridin-2-yl)-2,3-dihydroquinazolin-4(1H)-one* (**6b**). Off-white solid in 84% (Method A) or 96% (Method B) yield; mp 135–136 ^1^H-NMR (CDCl_3_): δ 1.39 (2H, m, CH_2_), 1.52 (4H, m, 2CH_2_), 2. 42 (5H, br.s, 2CH_2_ & CH), 2.61 (2H, m, CH_2_), 3.09 (1H, m, CH), 4.29 (2H, m, CH), 5.20 (1H, s, CH), 5.84 (1H, d, *J* = 2 Hz, NH), 6.51 (1H, d, *J* = 8 Hz, Ar), 6.77 (1H, t, *J* = 7.2 Hz, Ar), 7.17 (2H, td, *J* = 7.6, 1.2, 8, 4.4 Hz, Ar), 7.23 (1H, d, *J* = 8 Hz, Ar),7.57 (1H, td, *J* = 7.6, 1.6, 8, 1.6 Hz, Ar), 7.87 (1H, dd, *J* = 8, 1.6 Hz, Ar), 8.55 (1H, d, *J* = 4.4 Hz, Ar) ppm; ^13^C-NMR (CDCl_3_): δ 28.9, 30.5, 47.8, 59.4, 61.7, 77.0, 119.5, 121.2, 123.9, 124.8, 127.9, 132.9, 138.1, 141.6, 149.9, 154.5, 163.8, 167.8 (CON) ppm. LC/MS (ESI): 337.51 [M + H]^+^; Anal. for C_20_H_24_N_4_O; Calcd: C, 71.40; H, 7.19; N, 16.65; Found: C, 71.66; H, 7.41; N, 16.89.

*3-(2-Morpholinoethyl)quinazolin-4(3H)-one* (**7**). Off-white solid in 85% (Method A) or 97% (Method B) yield; mp 221–223 °C (Lit [46] 89% yield); ^1^H-NMR (CDCl_3_): δ 2.49 (4H, t, *J* = 4.4 Hz, CH_2_NCH_2_), 2.58 (2H, t, *J* = 6, 6.4 Hz, NCH_2_CH_2_N), 3.49 (2H, td, *J* = 6.8, 6.4, 5.2 Hz, NCH_2_CH_2_N), 3.69 (4H, td, *J* = 5.2, 10.4, 4.4, 4.8 Hz, CH_2_OCH_2_), 6.62–6.67 (3H, m, Ar), 7.17 (1H, td, *J* = 7.2, 1.6, 8, 1.2 Hz, Ar), 7.29 (1H, dd, *J* = 7.2, 1.6 Hz, Ar); ^13^C-NMR (CDCl_3_): δ 35.6, 53.3, 56.9, 66.9, 100.0, 116.1, 116.6, 117.3, 127.2, 132.2, 148.7, 169.29 (CON) ppm. LC/MS (ESI): 260.36 [M + H]^+^; Anal. for C_14_H_17_N_3_O_2_; Calcd: C, 64.85; H, 6.61; N, 16.20; Found: C, 64.99; H, 6.82; N, 16.47.

### 3.3. X-Ray Measurements

Compound **3a** was obtained as single crystals by slow evaporation at room temperature from dichloromethane–hexane (4:6) solution. Data were collected on a Bruker APEX-II D8 Venture area diffractometer equipped with graphite monochromatic Cu Kα radiation, λ = 1.54060 Å at 23 °C. Cell refinement and data reduction were carried out using Bruker SAINT. SHELXT [47,48] was used to solve the structure. The final refinement was carried out by full-matrix least-squares techniques with anisotropic thermal data for non-hydrogen atoms on *F*. CCDC 1896391 contains the supplementary crystallographic data for this compound and can be obtained free of charge from the Cambridge Crystallographic Data Centre via www.ccdc.cam.ac.uk/data_request/cif.

## 4. Conclusions

The present work described two simple protocols for the synthesis of 3-substituted-2,3-dihydro quinazolin-4(1*H*)-one and 3-(2-morpholinoethyl)quinazolin-4(3*H*)-one. The feature of this work is the use of 2-MeTHF, which offers both economical and environmentally friendly advantages over tetrahydrofuran for the synthesis of 2-amino-*N*-(2-substituted-ethyl)benzamide, considered a very important intermediate for the preparation of several derivatives with biological activities of interest [40,41]. In addition, microwave irradiation afforded the 3-substituted-2,3-dihydroquinazolin-4(1*H*)-one derivative final products in less reaction time and with higher yield and purity than conventional heating. NMR (^1^H and ^13^C) spectra, elemental analysis, and LC-MS confirmed the structures of all compounds obtained. 

Finally, this protocol could be a useful and attractive process for the synthesis of numerous 2,3-dihydroquinazolin-4(1*H*)-one derivatives of biological interest.

## Figures and Tables

**Table 1:** Reaction of isatoic anhydride using different solvents to afford the product **3a**.

Entry	Solvent	Time (h)	Yield (%)	Mp (°C)
**1**	Water–acetone	8	71	104–106
**2**	THF	5–6	86	113–114
**3**	2-MeTHF	4–5	95	113–114

**Table 2:** Experimental details of **3a**.

**Crystal Data**	
Chemical formula	C_13_H_19_N_3_O_2_
Mr	249.31
Crystal system, space group	Monoclinic, *P*2_1_/*c*
Temperature (K)	296
*a*, *b*, *c* (Å)	13.6879 (11), 10.2118 (9), 9.7884 (9)
β (°)	105.068 (7)
V (Å^3^)	1321.2 (2)
*Z*	4
Radiation type	Cu *K*α
µ (mm^−1^)	0.70
Crystal size (mm)	0.51 × 0.45 × 0.11
**Data collection**	
Diffractometer	Bruker APEX-II D8 venture diffractometer
Absorption correction	Multi-scan SADABS Bruker 2014
T_min_, T_max_	0.887, 0.912
No. of measured, independent, and observed [I > 2σ(I)] reflections	12783, 2298, 1393
R_int_	0.088
**Refinement**	
R[*F^2^* > 2σ( *F^2^*)], wR(*F^2^*), S	0.052, 0.152, 1.02
No. of reflections	2298
No. of parameters	176
No. of restraints	H atoms treated by a mixture of independent and constrained refinement
H-atom treatment	0.21, −0.19
Δρ_max_, Δρ_min_ (e Å^−3^)	0.052, 0.152, 1.02

**Table 3:** Yield (%), reaction time (h, min), and mp (°C) of 3-substituted-2,3-dihydroquinazolin-4(1*H*)-one derivatives.

Compound No.	R_1_	R_2_	R_3_	Reaction Time (h), Yield% Conventional Heating	Reaction Time (min), Yield% Microwave Irradiation	Mp (°C)
**4a**	H	H	H	4(86)	6(92)	142–144
**4b**	H	Br	H	4(87)	6(96)	133–135
**4c**	H	Cl	H	4(84)	6(96)	165–166
**4d**	H	CH_3_	H	5(83)	6(96)	132–134
**4e**	H	NMe_2_	H	5(87)	7(96)	183–184
**4f**	OCH_3_	OCH_3_	OCH_3_	5(86)	7(97)	148–150
**5a**	H	H	H	4(80)	6(93)	120–121
**5b**	H	Br	H	4(82)	6(97)	198–198
**5c**	H	OCH_3_	H	5(81)	6(94)	146–147
**6a**	H	H	H	4(83)	6(97)	154–155
**6b**	H	H	H	4(84)	6(96)	135–136
**7**	H	H	H	6(85)	8(97)	221–223

## Supplementary Materials

The following are available online.
