# Supplementary material for: A Simple, Efficient, and Eco-Friendly Method for the Preparation of 3-Substituted-2,3-dihydroquinazolin-4(1H)-one Derivatives
Source: Molecules. 2019 Nov 9;24(22):4052. doi: 10.3390/molecules24224052 (PMC6891463; doi:10.3390/molecules24224052)

A Simple, Efficient and Eco-friendly Method for Preparation of 3-Substituted-2,3-dihydroquinazolin-4(1H)-one Derivatives

Zainab Almarhoon, Kholood A. Dahlous, Hazem A. Ghabbour, Ayman El-Faham*

Table of Content

Selected geometric parameters (Å, °) for compound **3a**	**Table S1**
Hydrogen-bond geometry (Å, °) for compound **3a**	**Table S2**
^{1}H-NMR and ^{13}C-NMR for compound **3a**	**Figure S1**
^{1}H-NMR and ^{13}C-NMR for compound **3b**	**Figure S2**
^{1}H-NMR and ^{13}C-NMR for compound **4a**	**Figure S3**
^{1}H-NMR and ^{13}C-NMR for compound **4b**	**Figure S4**
^{1}H-NMR and ^{13}C-NMR for compound **4c**	**Figure S5**
^{1}H-NMR and ^{13}C-NMR for compound **4d**	**Figure S6**
^{1}H-NMR and ^{13}C-NMR for compound **4e**	**Figure S7**
^{1}H-NMR and ^{13}C-NMR for compound **4f**	**Figure S8**
^{1}H-NMR and ^{13}C-NMR for compound **5a**	**Figure S9**
^{1}H-NMR and ^{13}C-NMR for compound **5b**	**Figure S10**
^{1}H-NMR and ^{13}C-NMR for compound **5c**	**Figure S11**
^{1}H-NMR and ^{13}C-NMR for compound **6a**	**Figure S12**
^{1}H-NMR and ^{13}C-NMR for compound **6b**	**Figure S13**
^{1}H-NMR and ^{13}C-NMR for compound **7**	**Figure S14**

Table S1: Selected geometric parameters (Å, °) for compound **3a**

O1—C7	1.237 (3)	N2—C8	1.458 (3)
O2—C11	1.419 (4)	N3—C9	1.453 (3)
O2—C12	1.410 (4)	N3—C10	1.453 (4)
N1—C1	1.384 (3)	N3—C13	1.459 (4)
N2—C7	1.338 (3)		
C11—O2—C12	109.6 (2)	O1—C7—N2	120.2 (2)
C7—N2—C8	119.9 (2)	N2—C7—C6	118.6 (2)
C9—N3—C10	111.31 (19)	N2—C8—C9	113.13 (19)
C9—N3—C13	112.48 (18)	N3—C9—C8	112.14 (18)
C10—N3—C13	108.3 (2)	N3—C10—C11	111.0 (2)
N1—C1—C2	119.43 (19)	O2—C11—C10	111.5 (2)
N1—C1—C6	122.9 (2)	O2—C12—C13	111.9 (2)
O1—C7—C6	121.21 (18)	N3—C13—C12	110.1 (2)

Table S2: Hydrogen-bond geometry (Å, °) for compound **3a**

D*—H···*A	***D*—H**	**H···*A***	***D*···*A***	***D*—H···*A***
N2—H1N2···N1^{i}	0.90 (3)	2.22 (3)	3.105 (3)	167 (2)
N1—H1A···N3^{ii}	0.860	2.460	3.255 (3)	153.0
N1—H1B···O1	0.860	2.090	2.698 (3)	128.0
N1—H1B···O1^{ii}	0.860	2.300	3.037 (3)	144.0
C8—H8B···O1^{iii}	0.970	2.550	3.474 (3)	160.0

Symmetry codes: (i) *x*, −*y*+1/2, *z*+1/2; (ii) −*x*+1, −*y*+1, −*z*+1; (iii) −*x*+1, *y*−1/2, −*z*+3/2.

Figure S1: ^{1}H-NMR and ^{13}C-NMR for compound **3a**

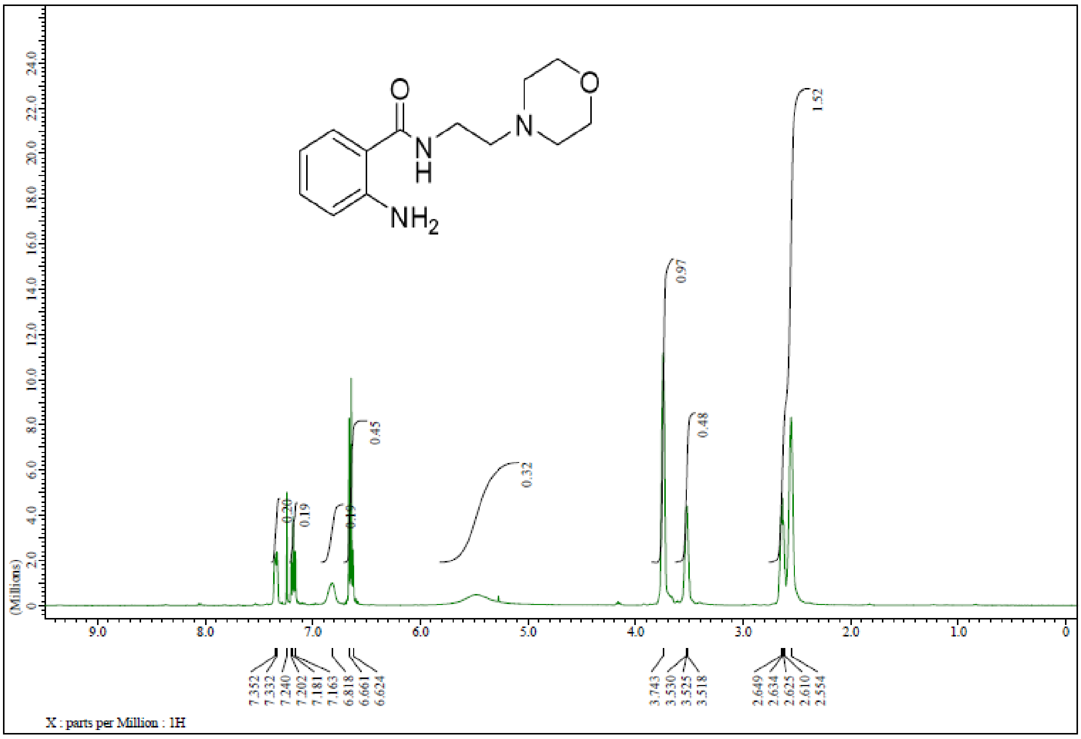

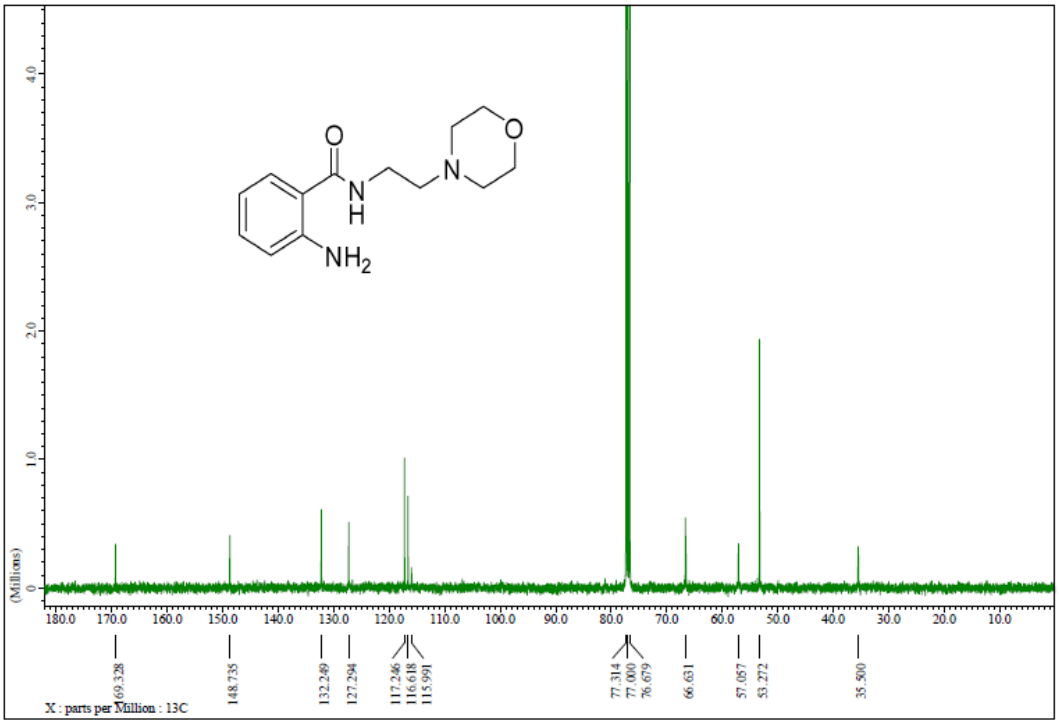

Figure S2: ^{1}H-NMR and ^{13}C-NMR for compound **3b**

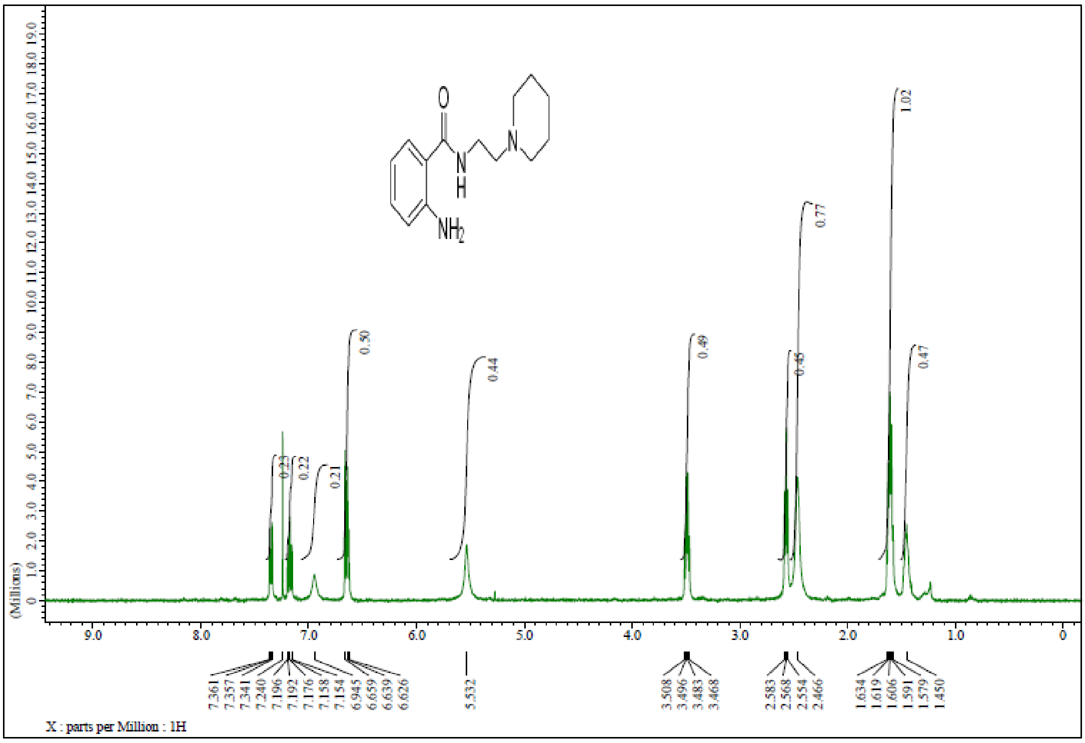

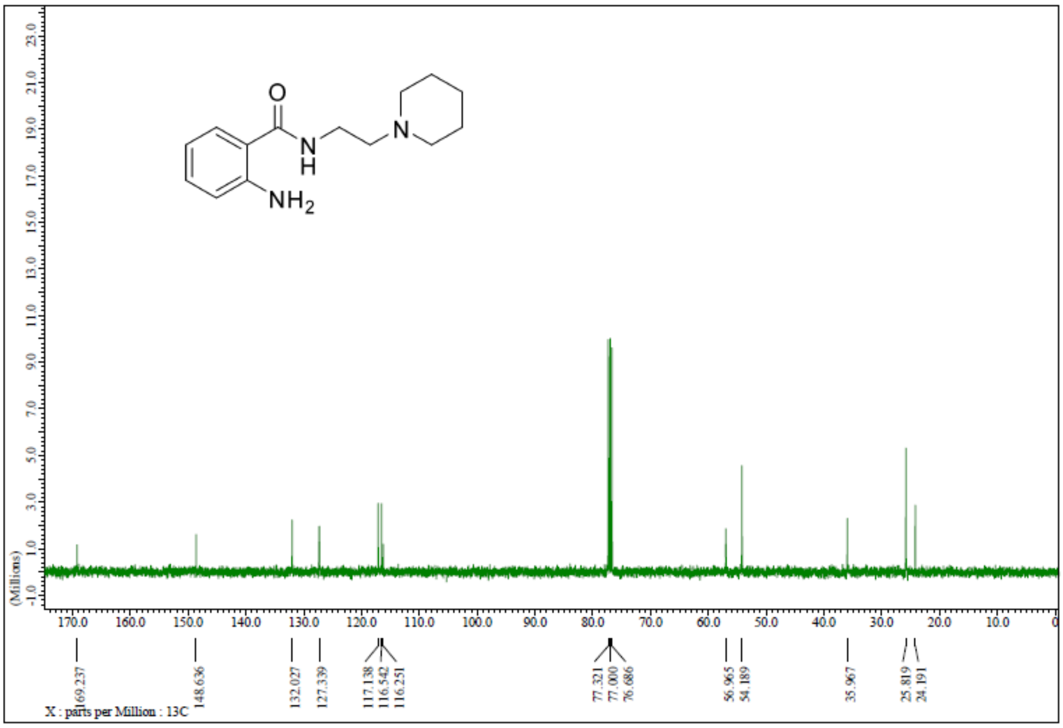

Figure S3: ^{1}H-NMR and ^{13}C-NMR for compound **4a**

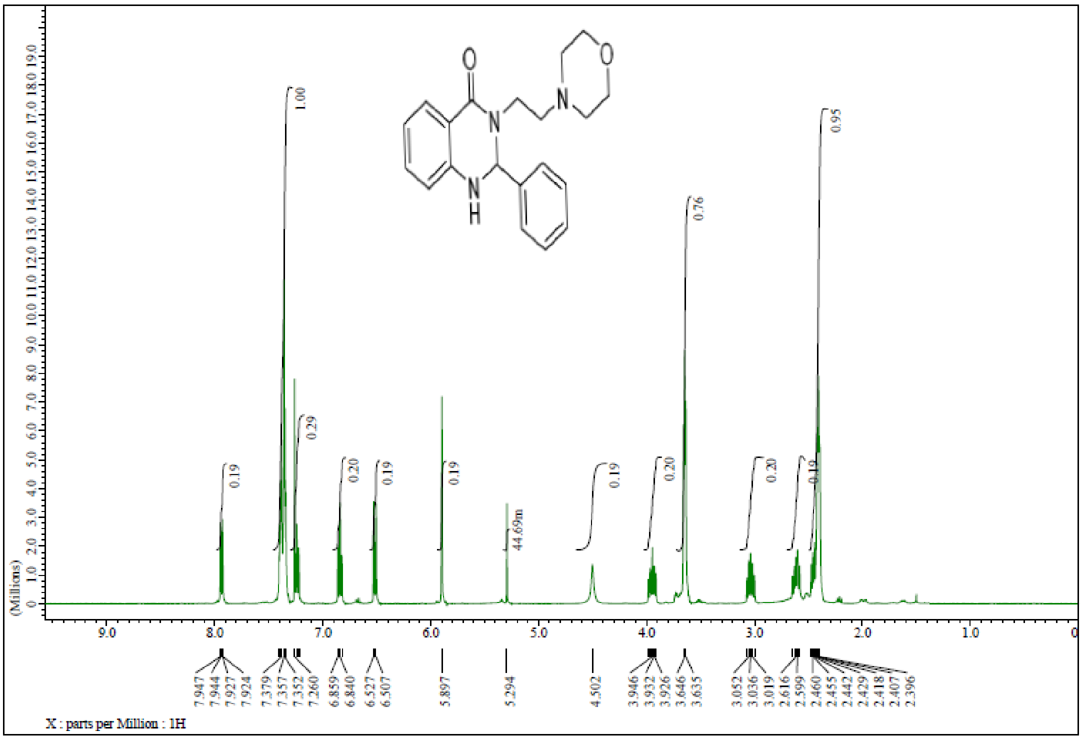

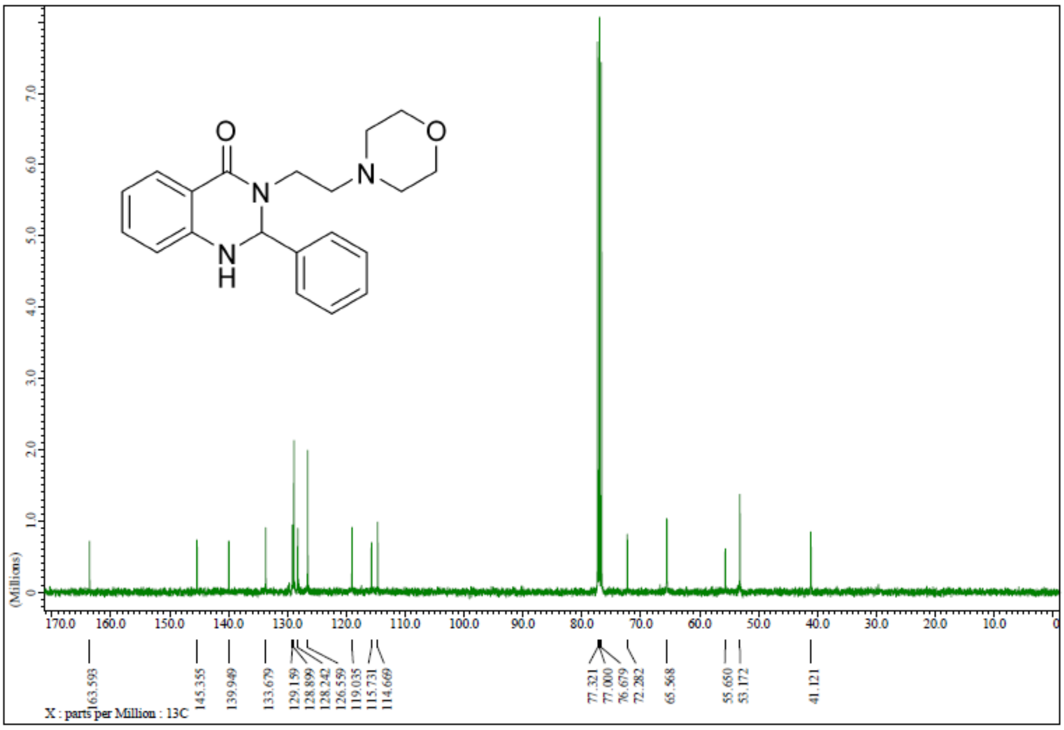

Figure S4: ^{1}H-NMR and ^{13}C-NMR for compound **4b**

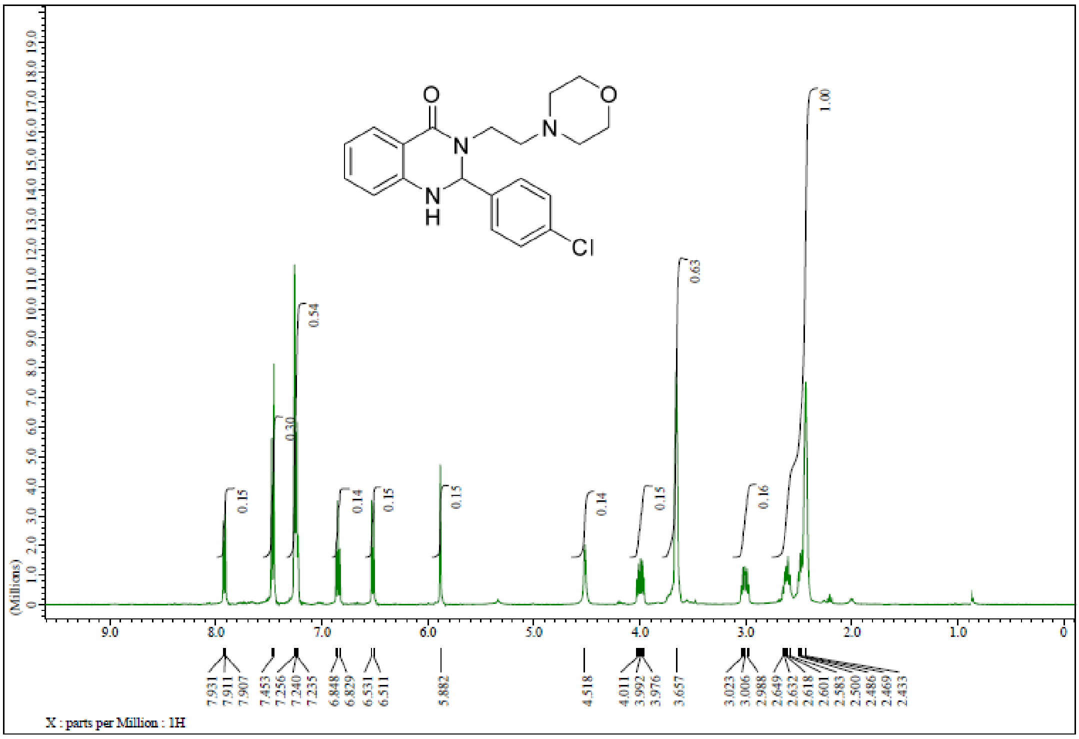

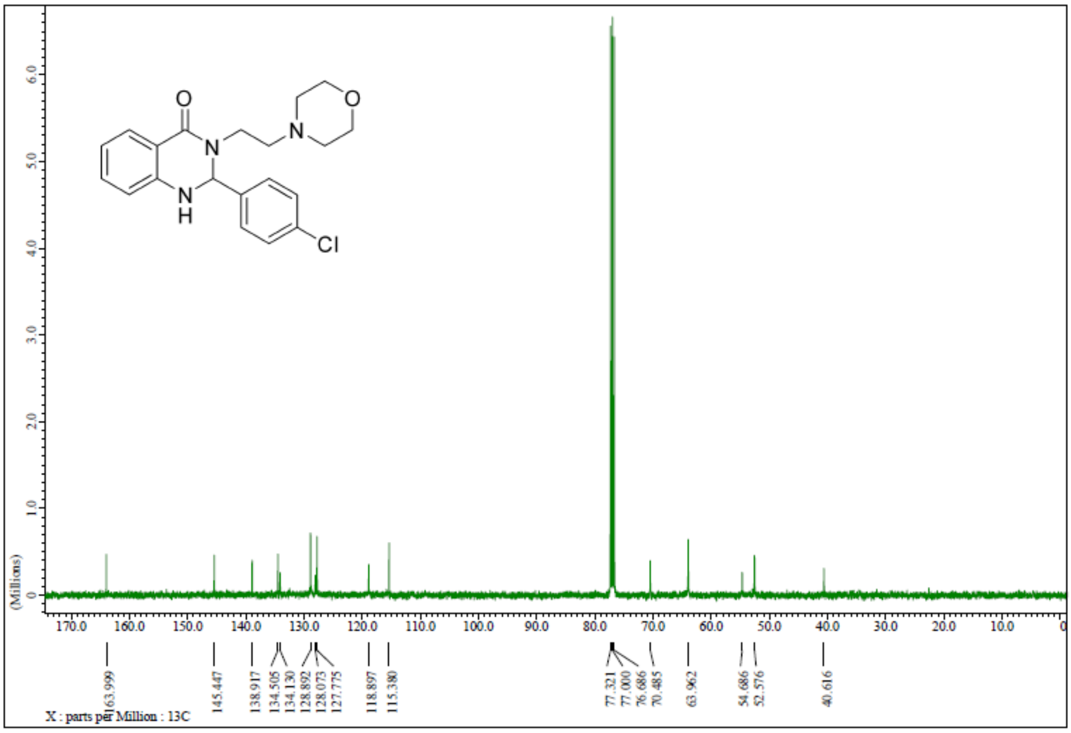

Figure S5: ^{1}H-NMR and ^{13}C-NMR for compound **4c**

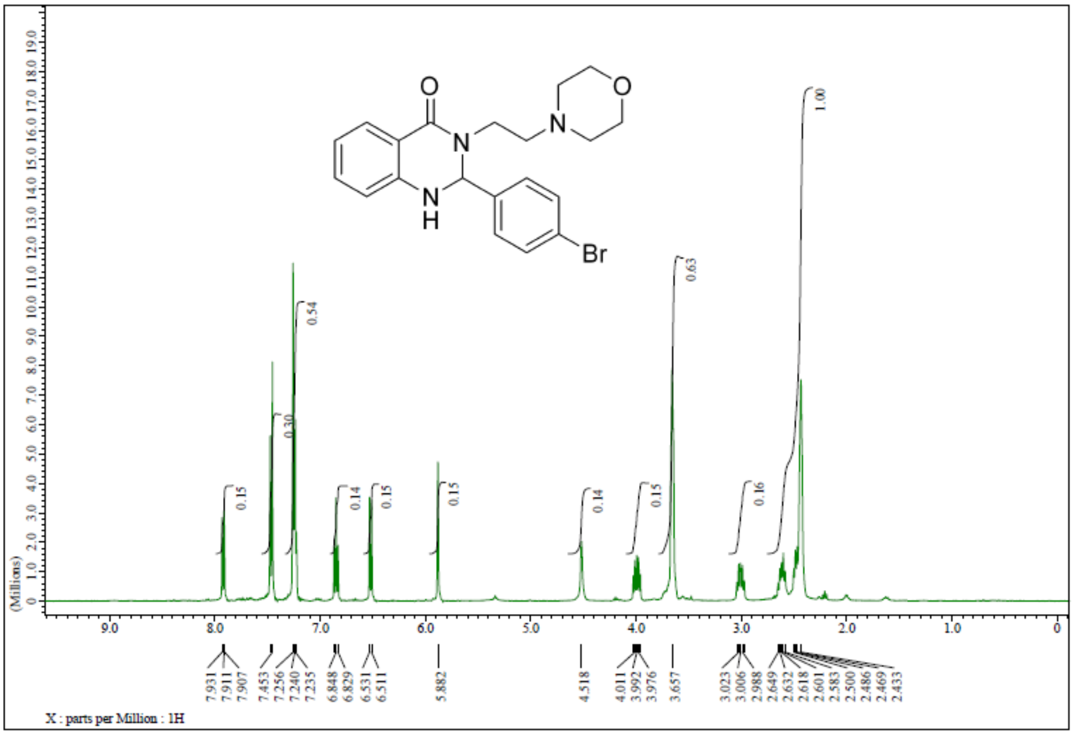

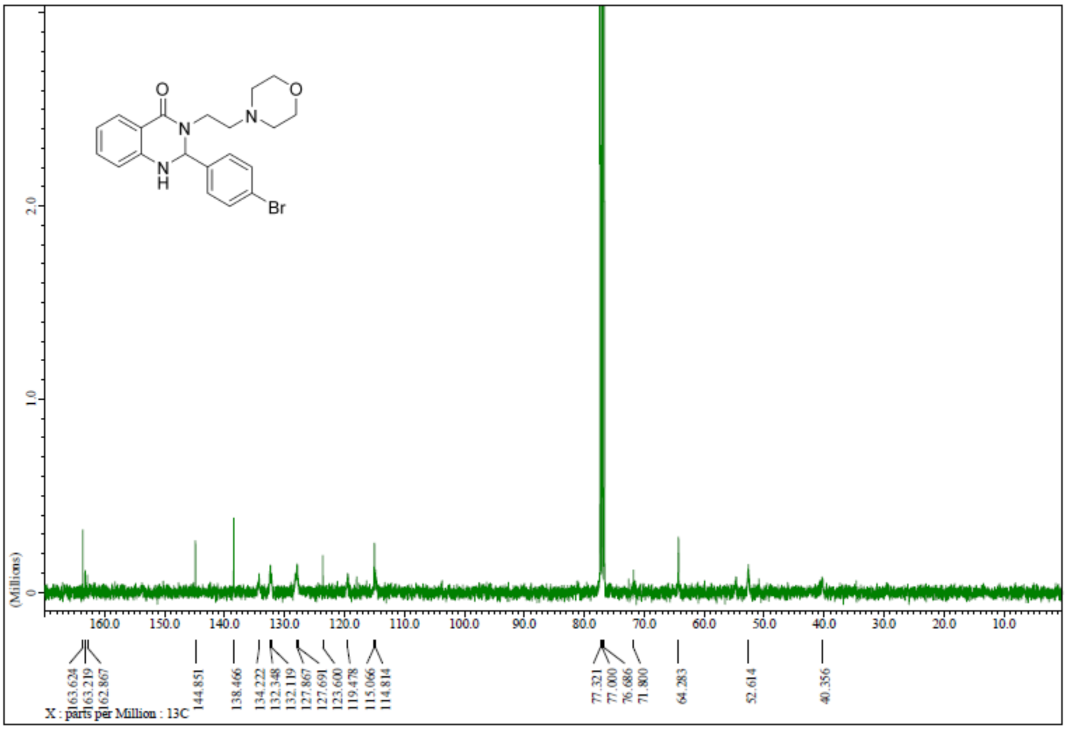

Figure S6: ^{1}H-NMR and ^{13}C-NMR for compound **4d**

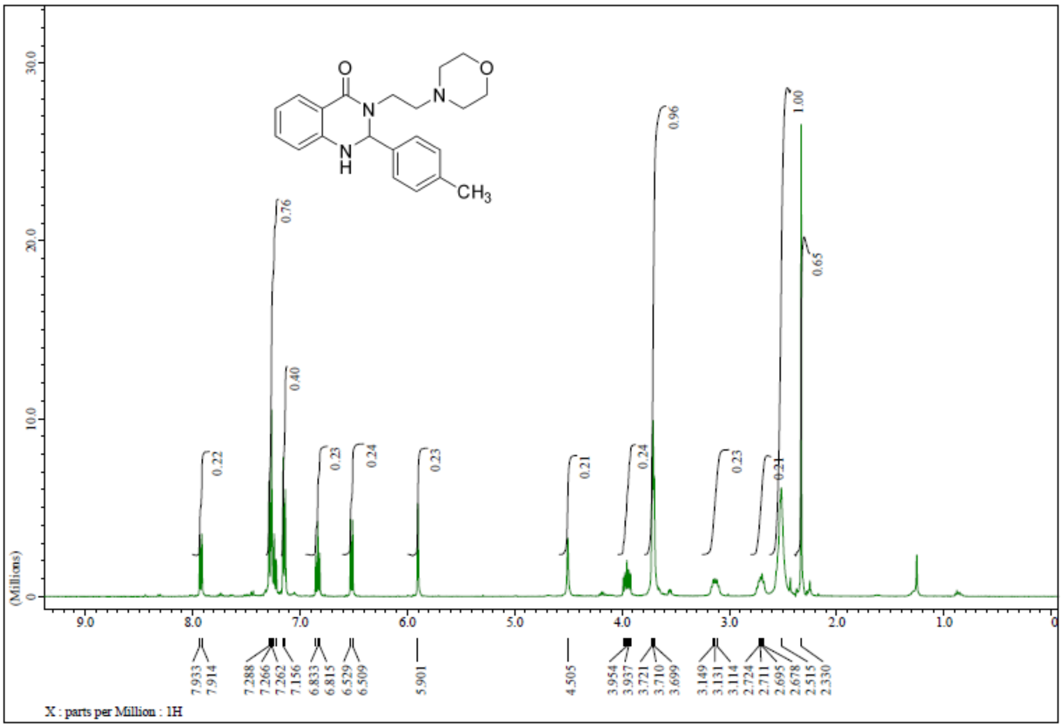

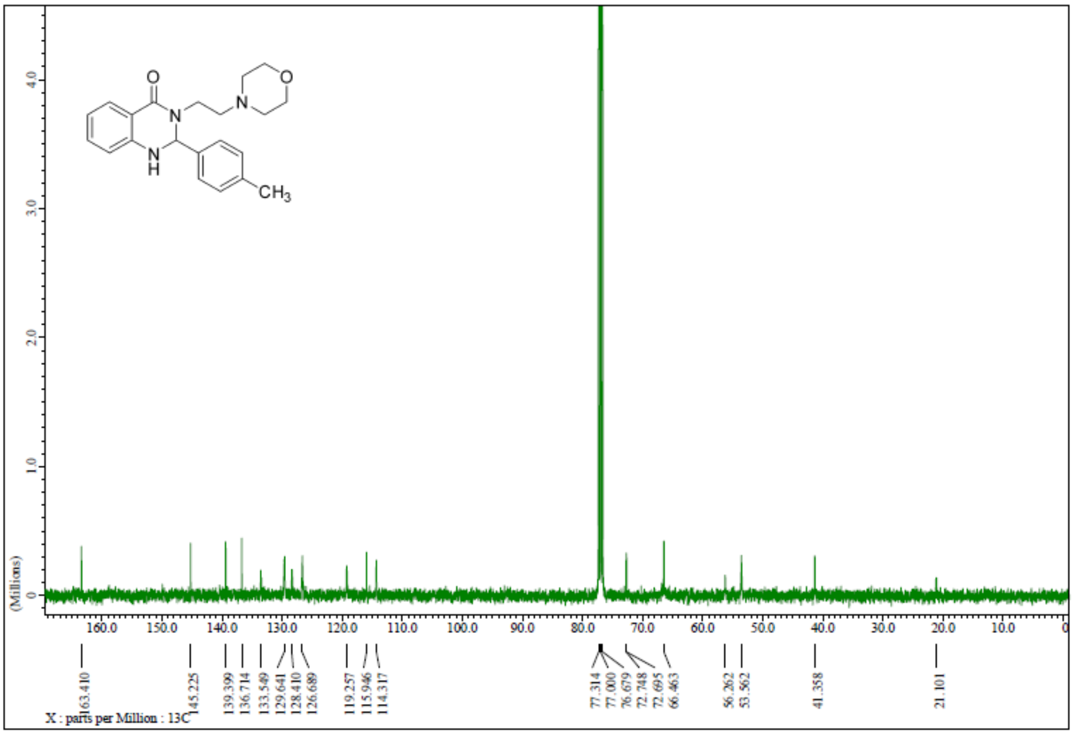

Figure S7: ^{1}H-NMR and ^{13}C-NMR for compound **4e**

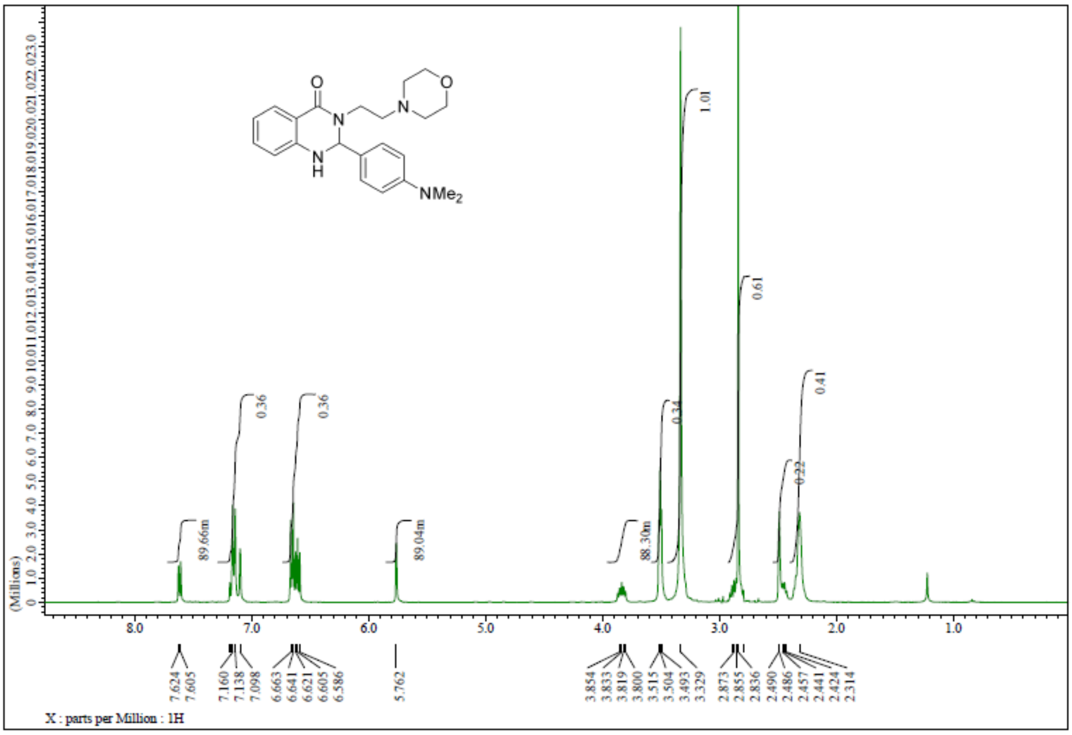

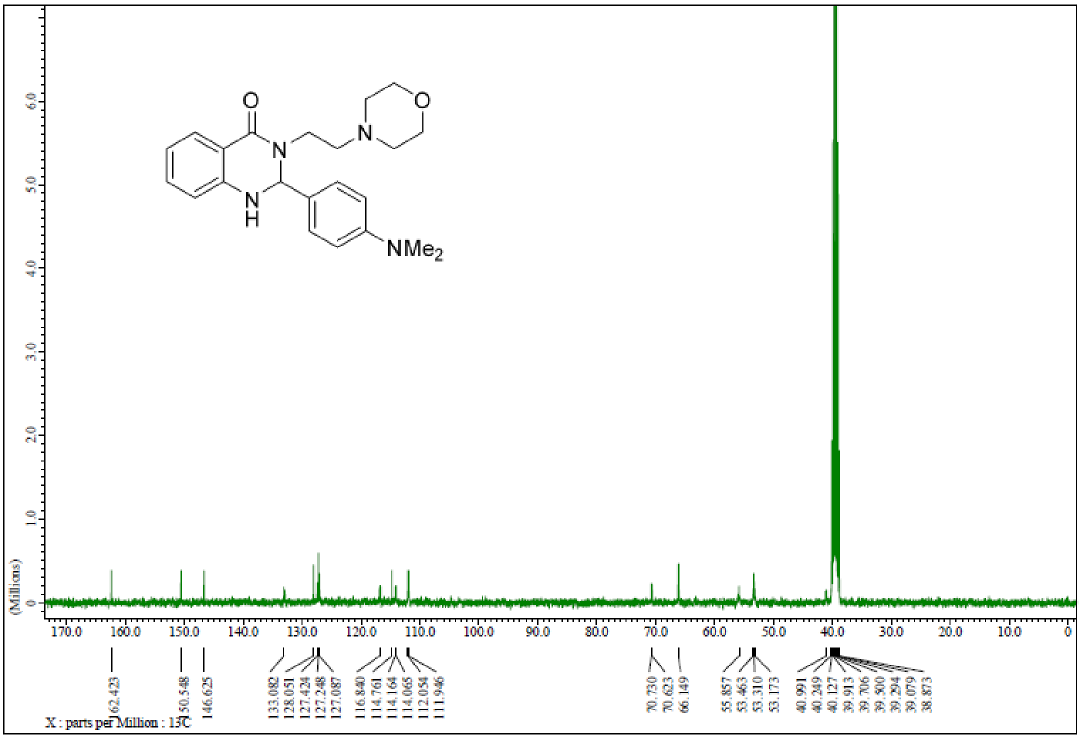

Figure S8: ^{1}H-NMR and ^{13}C-NMR for compound **4f**

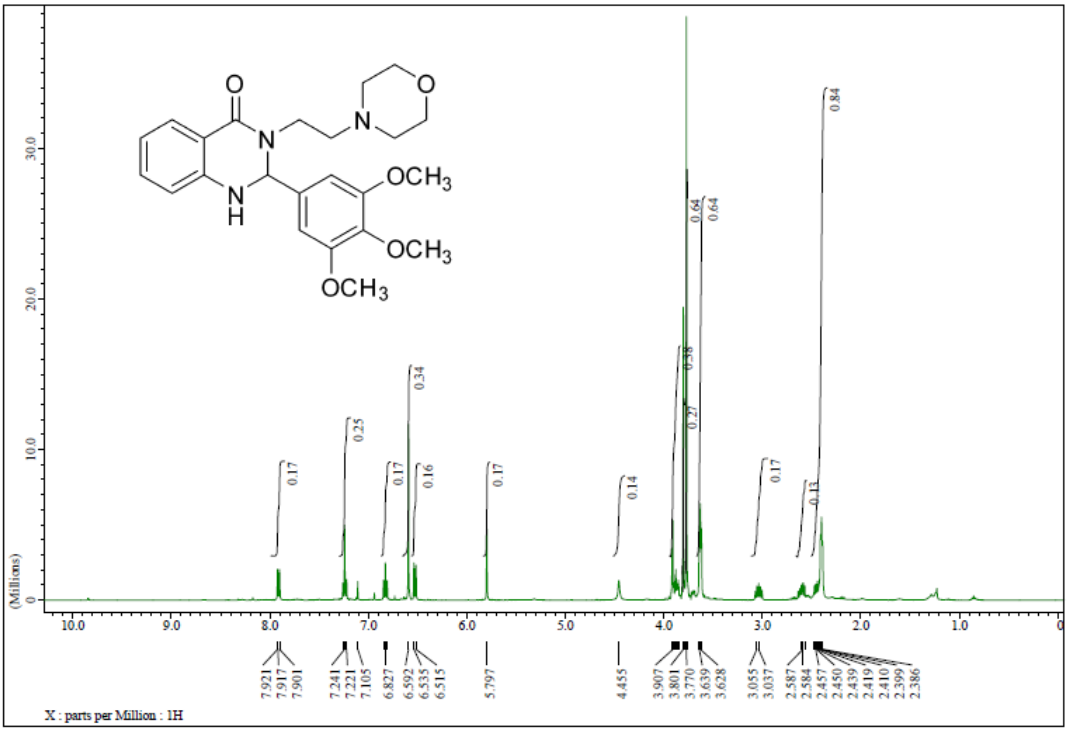

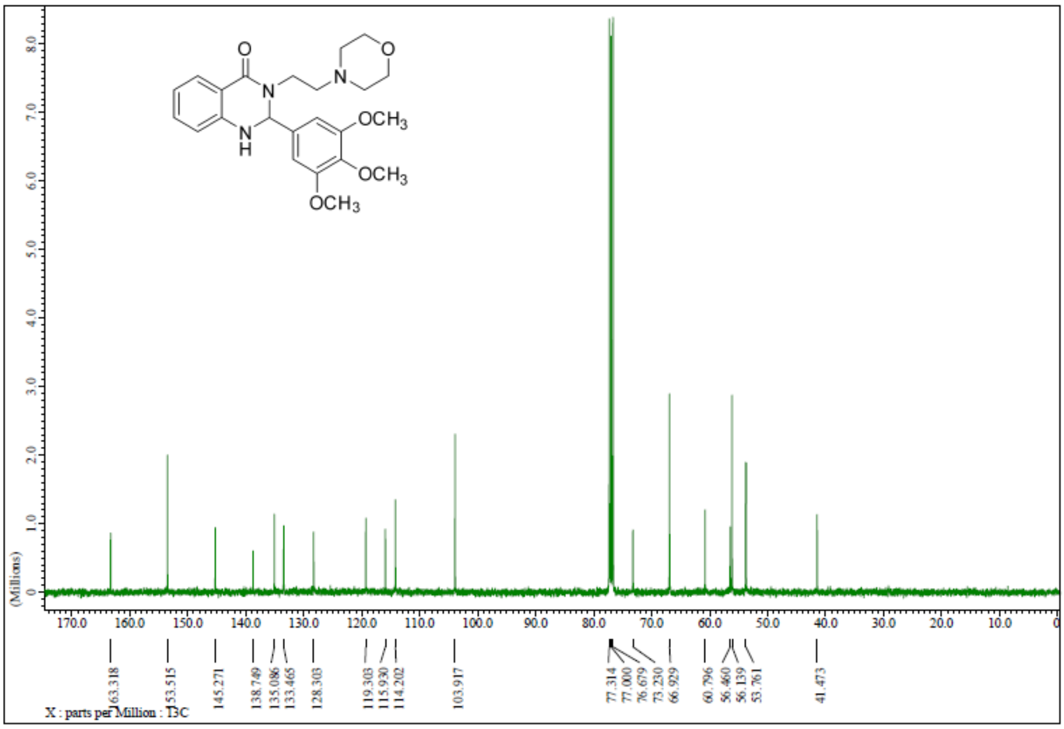

Figure S9: ^{1}H-NMR and ^{13}C-NMR for compound **5a**

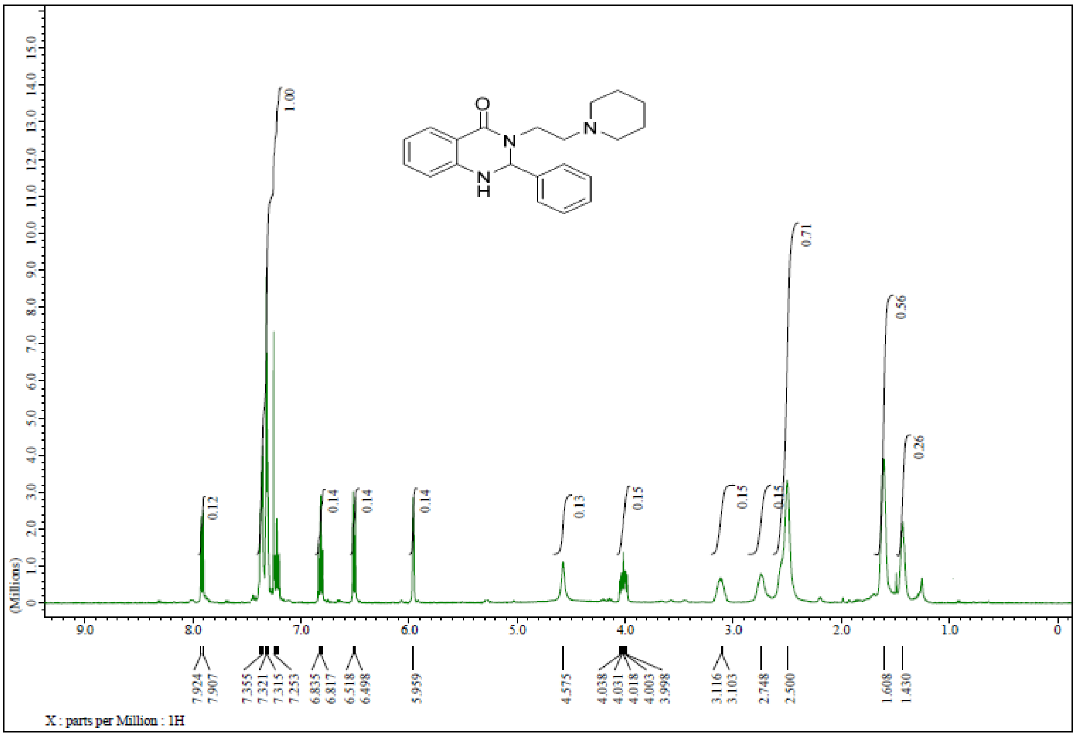

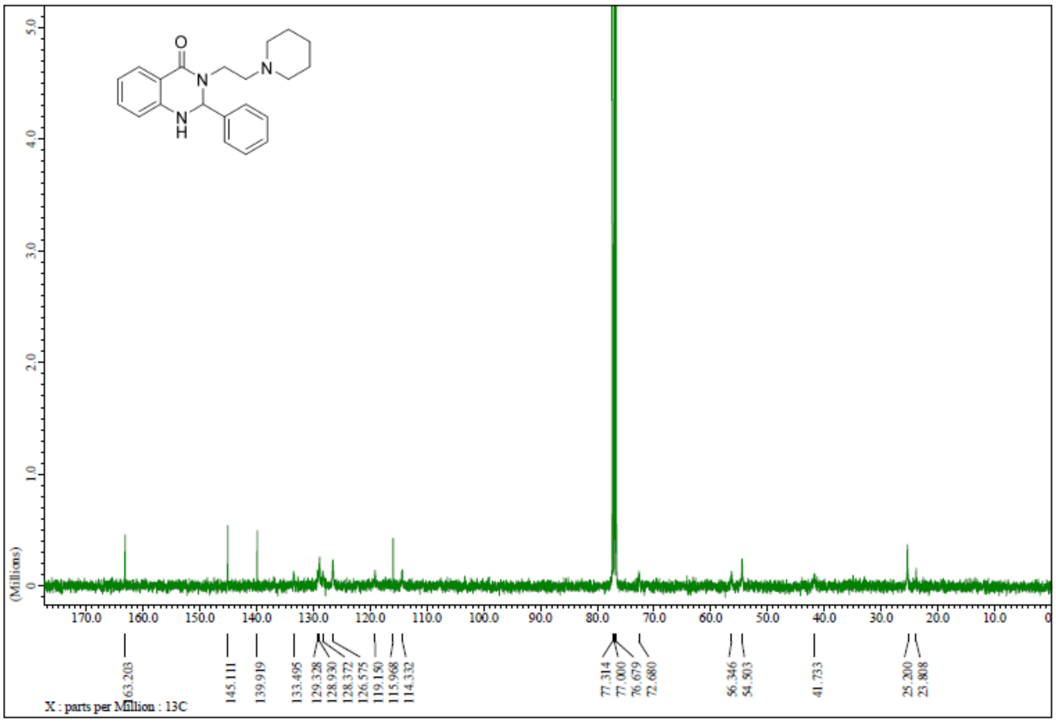

Figure S10: ^{1}H-NMR and ^{13}C-NMR for compound **5b**

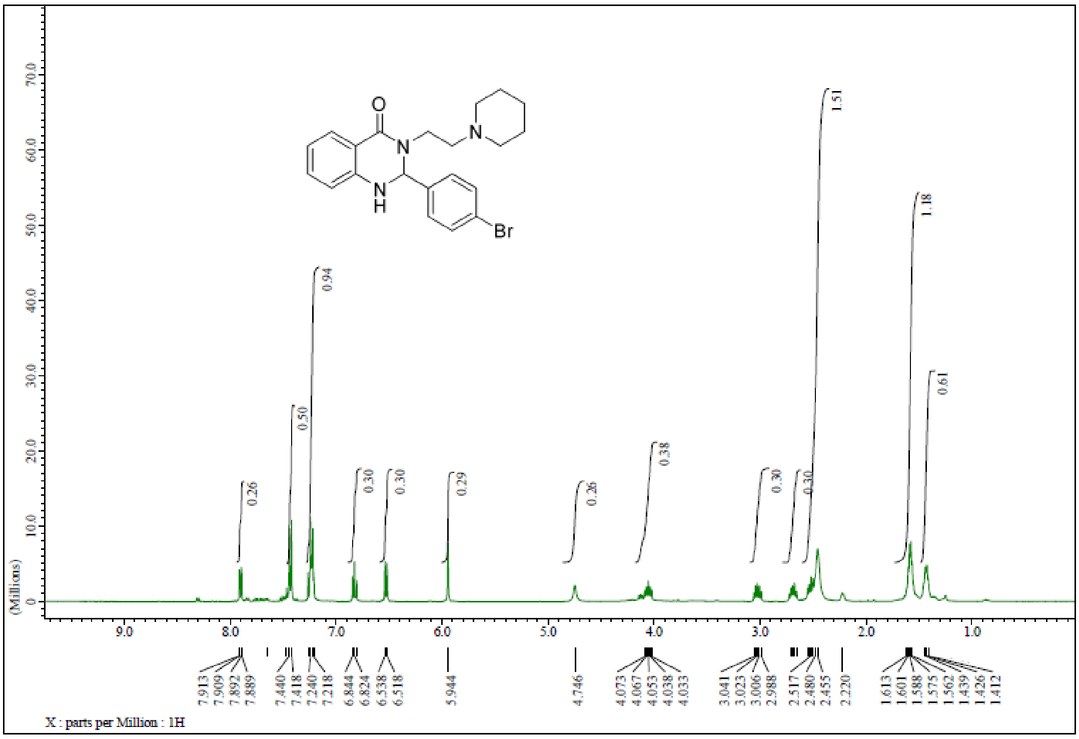

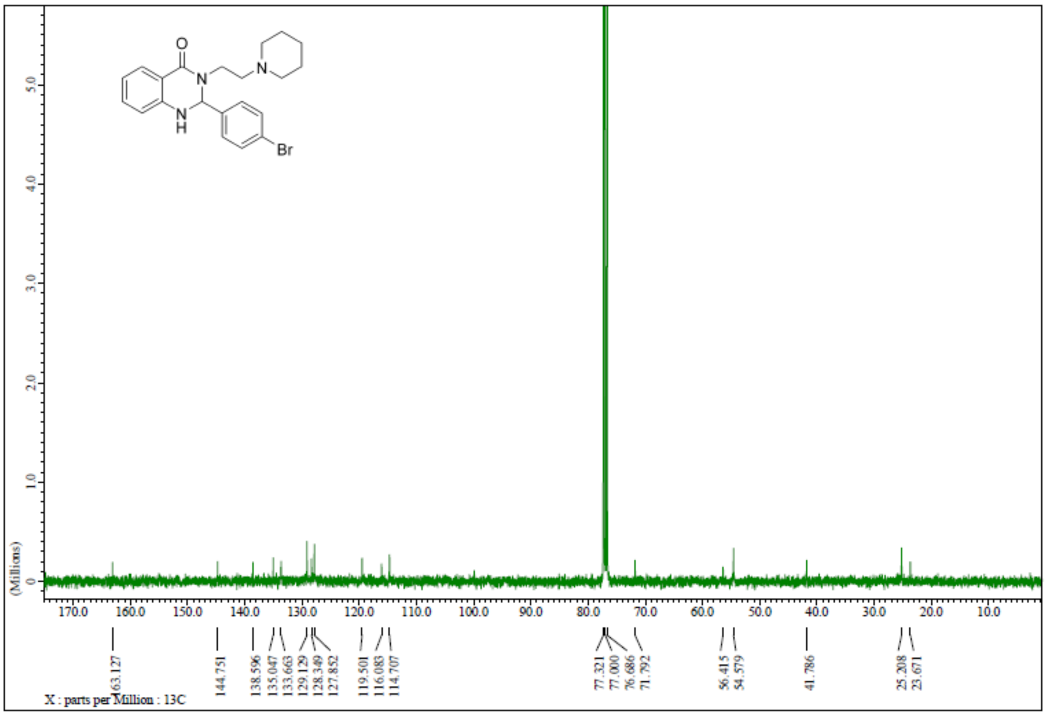

Figure S11: ^{1}H-NMR and ^{13}C-NMR for compound **5c**

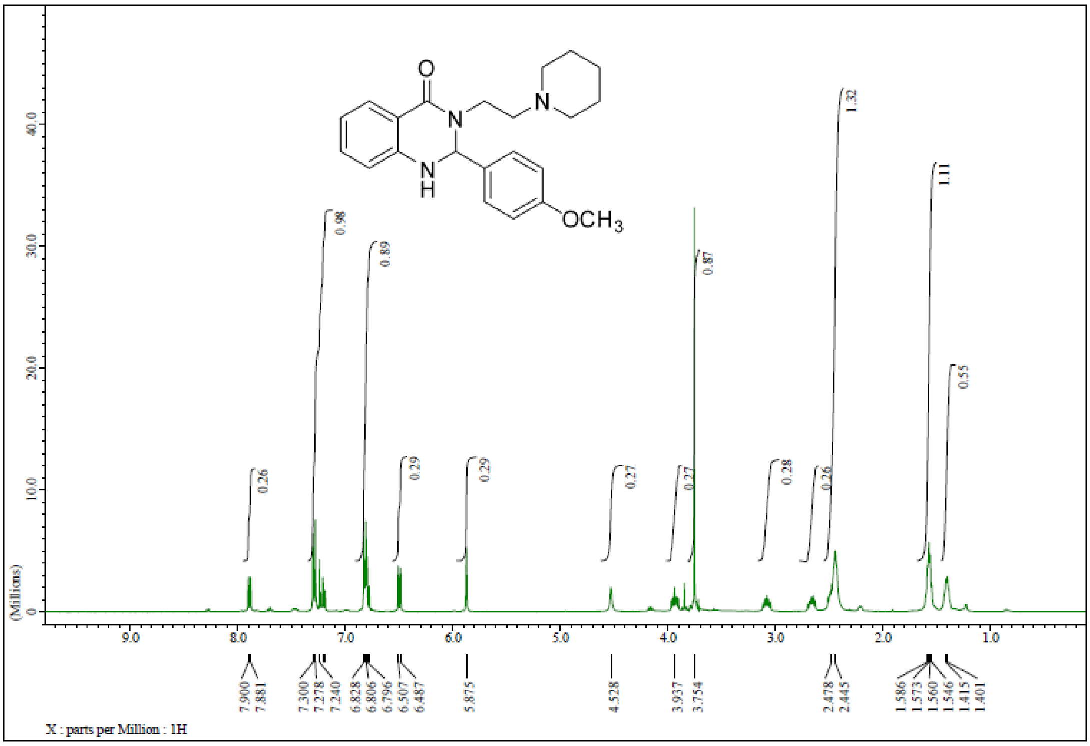

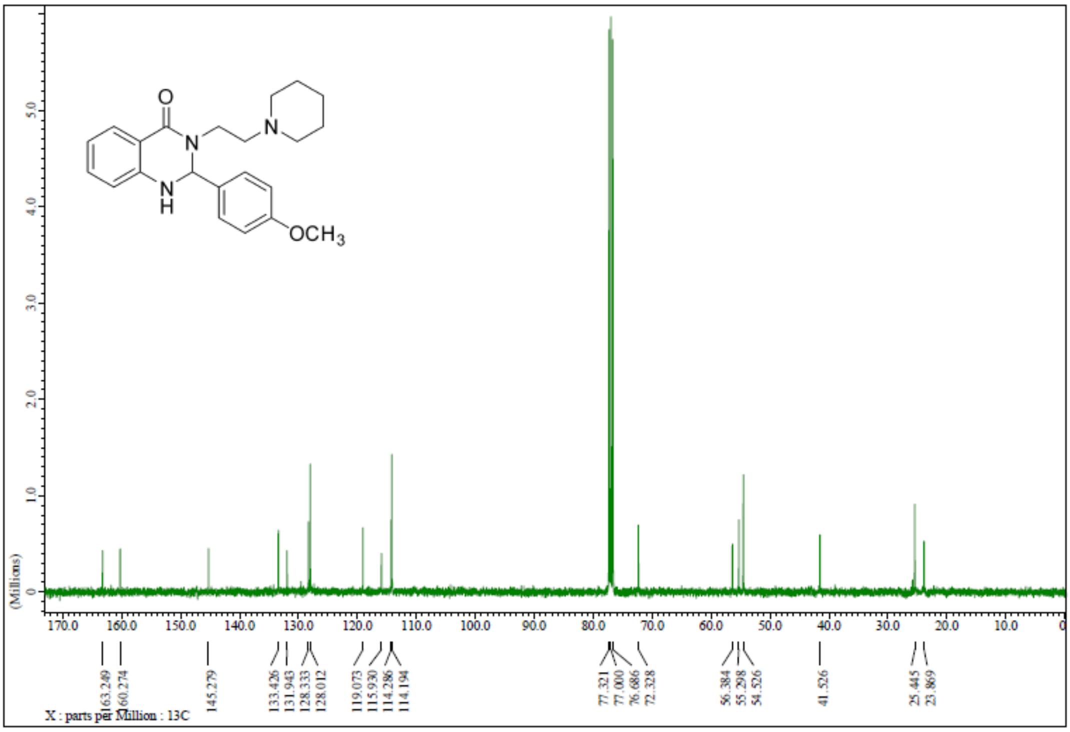

Figure S12: ^{1}H-NMR and ^{13}C-NMR for compound **6a**

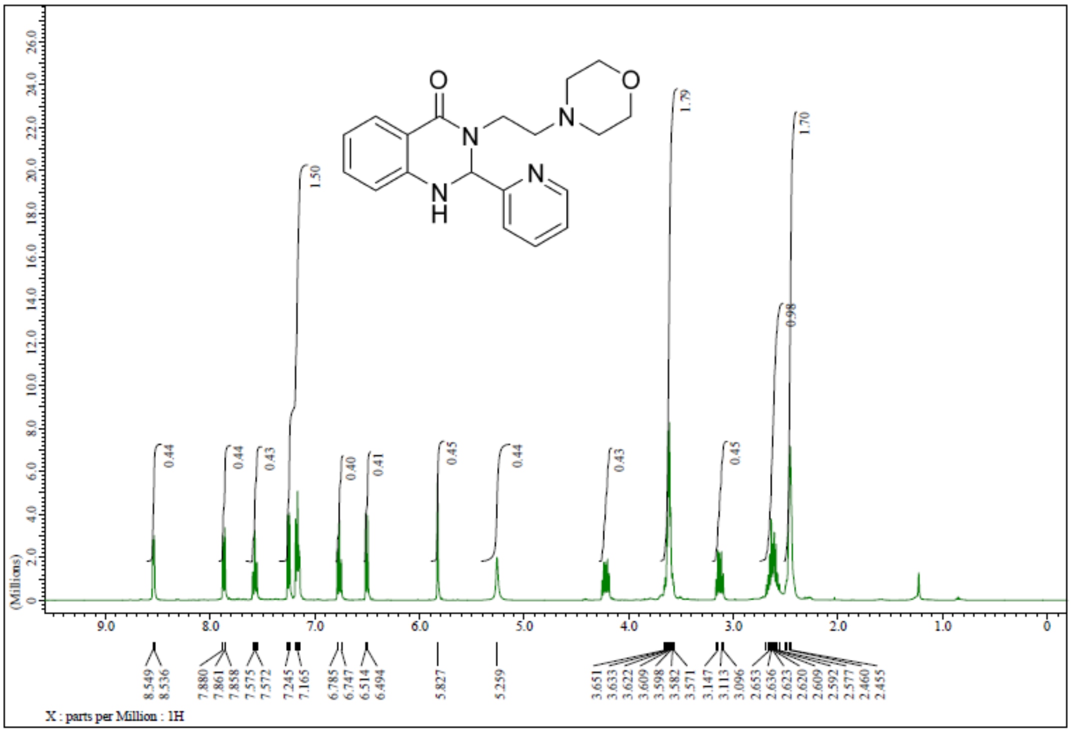

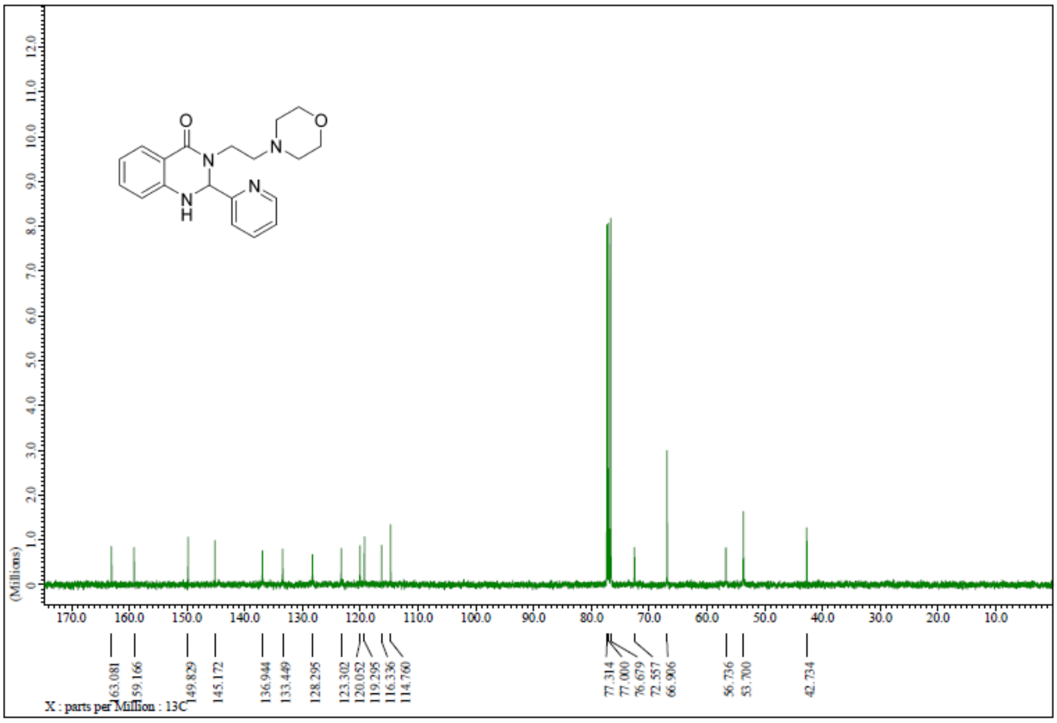

Figure S13: ^{1}H-NMR and ^{13}C-NMR for compound **6b**

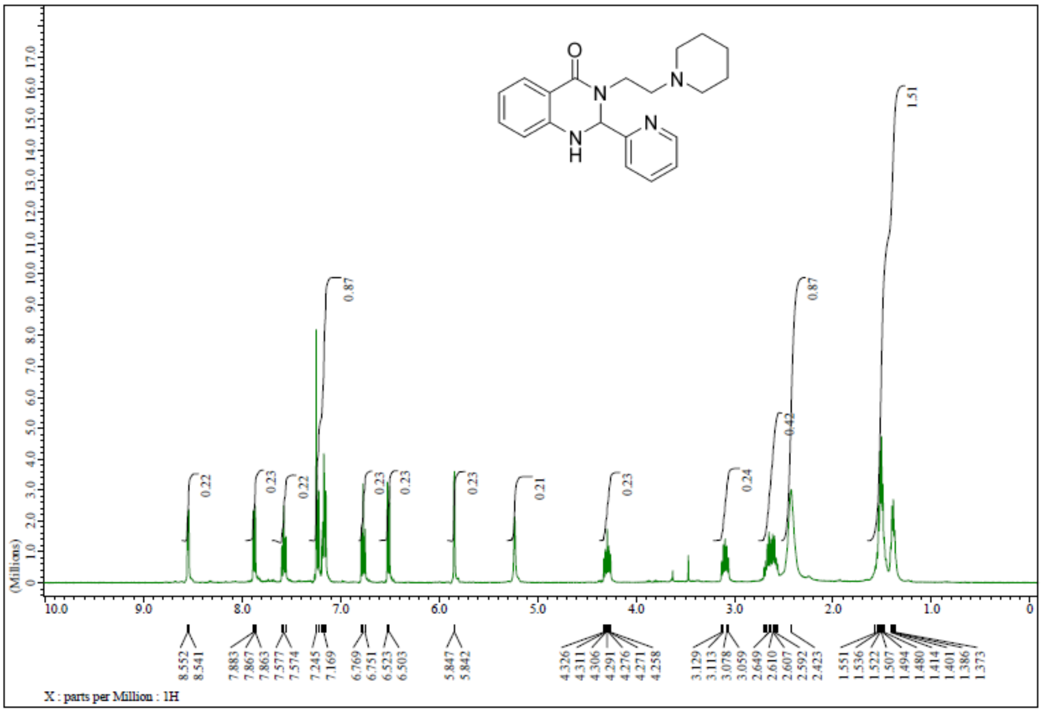

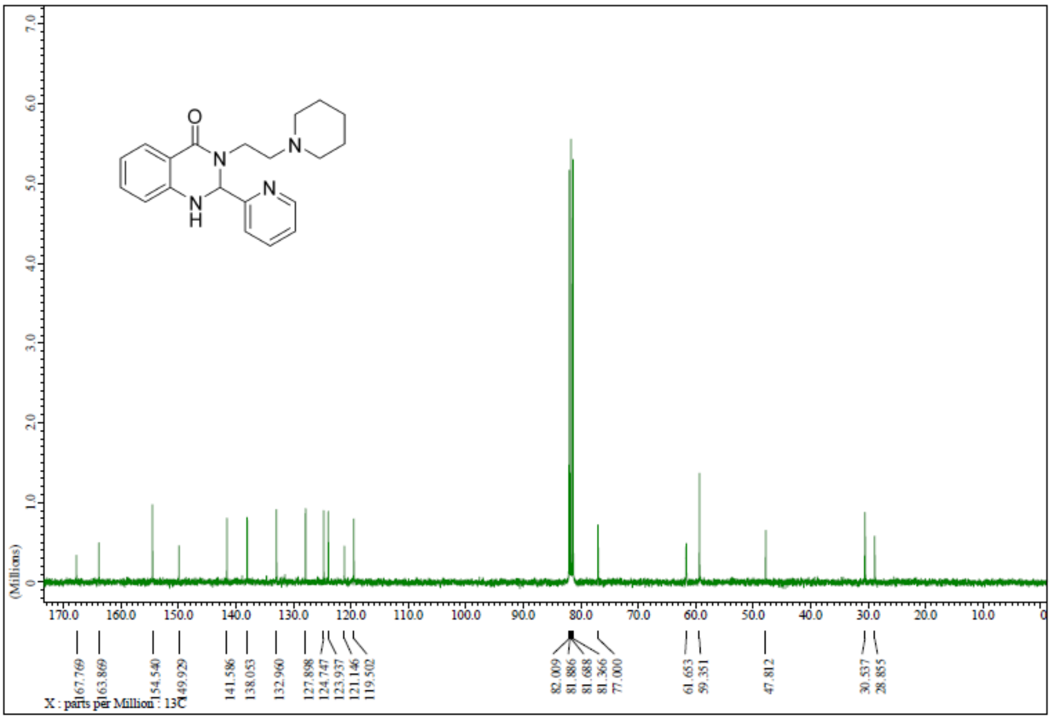

Figure S14: ^{1}H-NMR and ^{13}C-NMR for compound **7**

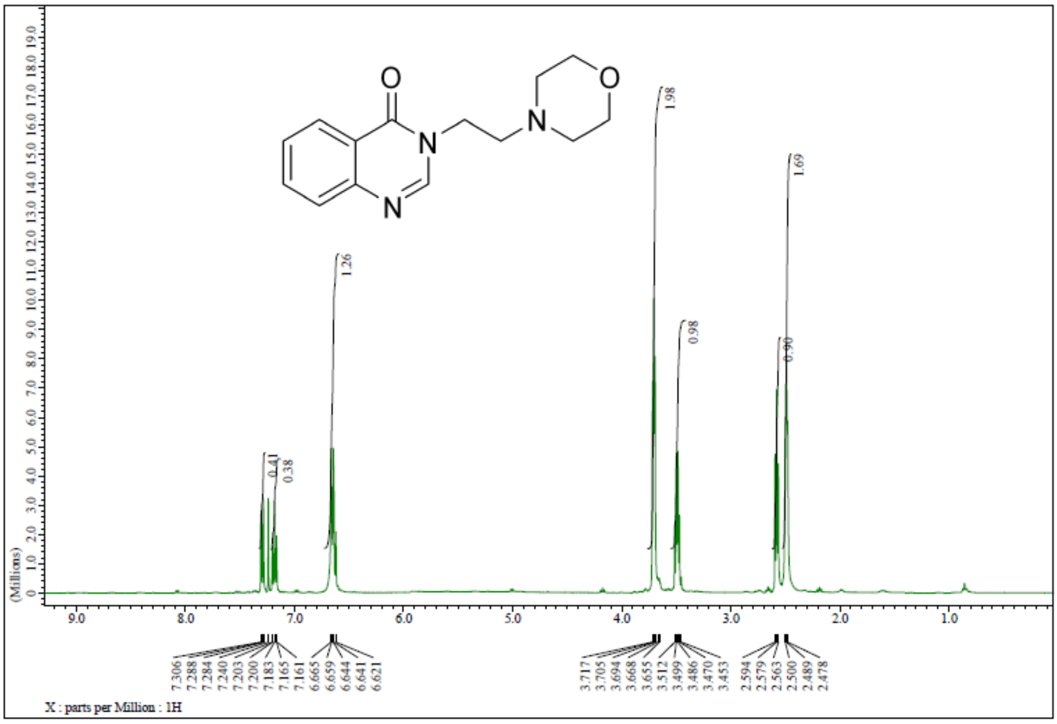

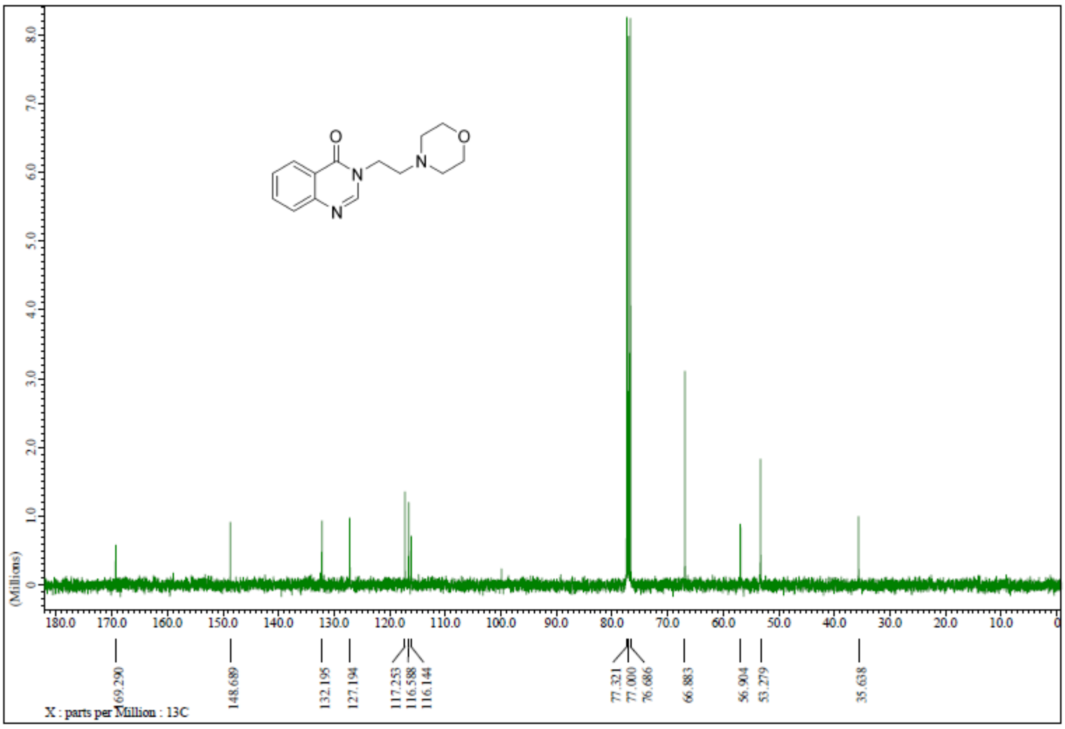

Supplement: Supplementary file 1 [file molecules-24-04052-s001.pdf]
